# Introducing an adjustable upper limb prosthesis into a Ugandan clinical service: impacts on free living behaviour and prosthetic use

**DOI:** 10.1038/s41598-025-96779-w

**Published:** 2025-04-04

**Authors:** N. Pickard, B. Mulindwa, L. Gracey-McMinn, M. Granat, A. Chadwell, S. Curtin, D. Howard, L. Ackers, R. Ssekitoleko, L. Kenney

**Affiliations:** 1 Centre for Human Movement and Rehabilitation Research, University of Salford, Salford, M6 6PU UK; 2 Biomedical Engineering Unit, Department of Physiology, Makerere University, Kampala, Uganda; 3 School of Healthcare Enterprise and Innovation, University of Southampton, Southampton, SO16 7NP UK; 4 Centre for Applied Health Research, University of Salford, Salford, M6 6PU UK

**Keywords:** Prosthetics, Upper-limb, Real-world behaviours, Appropriate technology, Uganda, Rehabilitation, Translational research

## Abstract

Access to prosthetic services in Uganda is poor, negatively impacting people with upper limb difference in function, community participation, and employment. Technologies to improve the efficiency and effectiveness of services are needed, but there is very little evidence of potential solutions. Off-the-shelf, adjustable prosthetic sockets, which can be fitted in minimal time without the complexities associated with traditional socket manufacturing, show promise. We tested the feasibility of introducing the Koalaa ALX, a prosthesis for people with trans-radial limb difference, and a peer-to-peer support system into a Ugandan clinical service. Prosthesis-worn and thigh-worn monitors successfully captured concurrent patterns of wear and free-living physical behaviours (including periods at home and away) at baseline, post-fitting, and six months post-fitting. End-of-study interviews captured participants’ experiences. Four of the eight participants had no previous experience using a prosthesis (new users). The ALX could be easily deployed and supported. All participants wore their ALX, with an increase in wear time among new users at six months. Prosthesis donning and doffing events were associated with behavioural changes (e.g., changes in stepping duration). The interviews revealed good levels of satisfaction, though concerns about access to repairs, cleaning, heat discomfort, and long-term support were noted.

## Introduction

The number of people living with traumatic limb loss in Sub-Saharan Africa has grown substantially from 1990 to 2019, in part due to the rapid increase in population^1^. Services are failing to meet existing demand, with the median level of access to prosthetics at 17% for the lower limb and 0% for the upper limb, according to a World Health Organisation report^2^. In the coming years, these already overstretched prosthetic services will likely experience increasing demands^3^.

This paper reports on a study conducted in Uganda that focused on upper limb prostheses. Comprehensive data on access to upper limb prostheses in Uganda are not available; however, people with limb differences face significant challenges in accessing prosthetic services^4–7^. The reasons for this include inadequate government funding, insufficient clinical facilities, lack of paid positions, and poorly functioning supply chains. Indeed, public sector orthopaedic workshops are provided with no budget to purchase components or essential consumables. This lack of purchasing power in the sector means there are few suppliers of components or materials, and prices are high. Donor priorities and other drivers mean that the limited resources in orthopaedic workshops tend to focus on providing services for people with lower limb difference^4^. For patients with a prosthesis, repair services are ad-hoc at best and are greatly hampered by issues similar to those mentioned above^8^.

Upper limb difference not only reduces function, but also impacts community participation^9^. Previously, we interviewed 17 people with upper limb difference living in Uganda, only three of whom had ever used a prosthesis and only one who owned a prosthesis at the time of the interview^10^. Some people reported voluntarily avoiding some social situations and/or covering up their limb difference, partly due to the stigma associated with limb difference. Performing activities of daily living outside of the home environment, where they could rely on the support of family and friends, was viewed as challenging. Finally, several interviewees reported losing their jobs due to their limb difference and finding alternative employment difficult.

One major bottleneck in almost all prosthetic services is the method used to fabricate prosthetic sockets. The vast majority of sockets are bespoke, fabricated in specialised facilities, and the quality of fit of the socket is dependent on the skill of the prosthetist. Manufacturing is a time-consuming process that requires access to specialised equipment and an appropriate stock of various materials. In government-funded workshops in Uganda, the necessary combination of skilled staff, well-maintained specialised equipment, and stocks of the (imported) materials needed to produce a comfortable socket is rarely present^11^. If a socket does not fit comfortably the first time, minor adjustments may be possible, but in some cases, a completely new socket would need to be fabricated.

An innovation that may offer prosthetists a more efficient way of delivering upper limb prosthetic services is adjustable sockets^12^. A few such designs are supplied ‘off the shelf,’ requiring only minor adjustments by a prosthetist/orthopaedic technologist to be appropriately sized for amputees of various residual limb diameters and lengths. This eliminates the need for the materials and specialised machinery needed to fabricate traditional sockets and potentially offers significant time savings to clinicians. As upper limb prosthesis users consistently report comfort as an area that needs addressing^13^, sockets that allow the user to make minor adjustments to fit on a day-to-day basis and are easy to don and doff may offer advantages to patients. Finally, off-the-shelf adjustable sockets may be easier to repair due to their modular design.

One such device for people with trans-radial limb difference is produced by Koalaa (Koalaa Ltd, London, England, NW10 6HF). In this study, we explored the feasibility of delivering the Koalaa ALX system, together with an associated support package, via a Ugandan public sector prosthetics service at Fort Portal Regional Referral Hospital (FPRRH). At the start of the study, it was unknown whether this device would be acceptable to patients in the Ugandan context and, if so, whether it may impact some of the negative consequences of poorly or unaddressed limb difference, as highlighted in our earlier work. The aims were:

1. To set up a pilot service to deliver Koalaa adjustable upper limb prosthetics at Fort Portal Regional Referral Hospital.
2. To test the feasibility of delivering a training and support package from both technologists’ and users’ perspectives and explore the extent to which patients take advantage of the support offered, including repairs.
3. To collect pilot data on the impact of the package on real-world use of prostheses and patient patterns of activity inside and outside of their home.

## Methods

Study setting. The study interventions primarily took place in the orthopaedic workshop at Fort Portal Regional Referral Hospital (FPRRH), Kabarole district, in Uganda. In the year before the study began, the workshop received a significant boost in its resources through a public–private partnership between FPRRH and Knowledge for Change, a UK- and Uganda-based non-governmental organisation (NGO). This led to a step change in the workshop’s productivity, details of which are provided elsewhere^14^.

Ethical approvals. Ethical approval for this study was granted by the University of Salford Research Ethics Committee (ref.8274), Makerere University School of Biomedical Sciences Research Ethics Committee (ref.SBS 2022–205), and the Uganda National Council for Science and Technology (ref.SIR192ES). Informed consent was gained from all participants, indicated using either a signature or thumbprint. All research was performed in accordance with relevant guidelines/regulations, and in accordance with the Declaration of Helsinki.

**Fig. 1:**
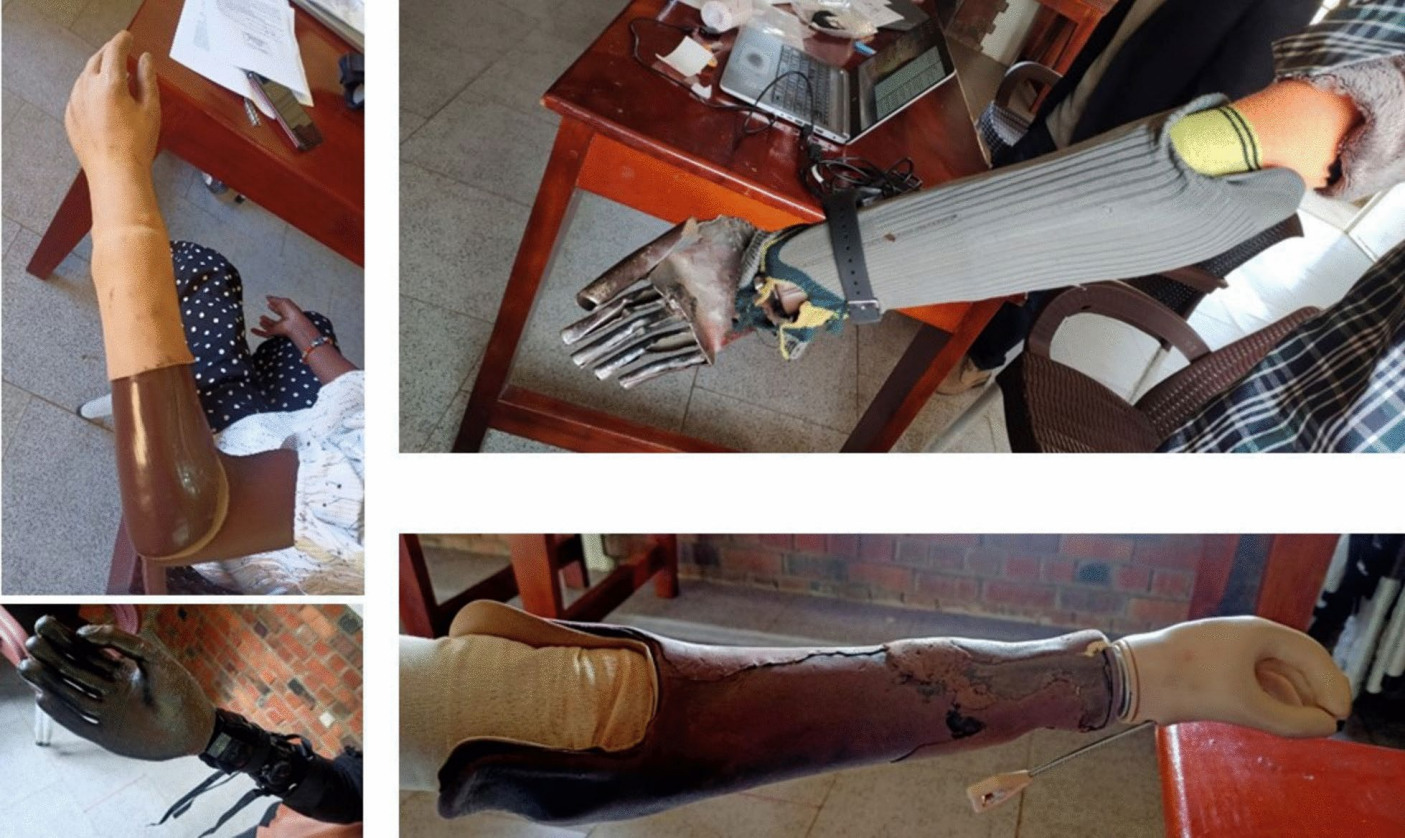
Prostheses used by the four participants who entered the study with an existing prosthesis.

Participants**.** Eight participants (6 males and 2 females, aged 24–42) with acquired unilateral limb difference at the trans-radial level were recruited (Table 1). The participants reported losing their limbs between 2 and 24 years before the study from a range of traumatic causes, including violence and industrial and road traffic accidents. Four participants owned a prosthesis, as shown in Fig. 1, and four had no previous experience using a prosthesis. One participant (P2) also presented with a below-knee amputation. The participants were identified from the list of patients at the FPRRH orthopaedic workshop and via word-of-mouth through existing contacts with upper limb difference established in a previous study.

**Table 1:** Participant demographics.

Participant number	Age range (years)	Gender	Affected side	Time since amputation (years)	Cause of amputation	Previous experience of using prostheses (number of years)?	Occupation
P1	20–30	M	L	14	Violence (mutilation)	Yes (12)	Self-employed
P2	20–30	F	L	24	Fire burn accident	Yes (7)	Student
P3	20–30	M	R	5	Road traffic accident	Yes (1)	Self-employed
P4	30–40	M	L	8	Industrial machine Injury	No	Farming
P5	20–30	F	L	2	Violence (domestic)	No	Unemployed
P6	20–30	M	R	5	Road traffic accident (boda[motorbike])	No	Unemployed
P7	40–50	M	R	6	Violence (robbery)	No	Farming
P8	30–40	M	R	11	Electric shock	Yes (11)	Mechanic

Previous work^15^ confirmed the potential of using mobile phones to offer guidance and support to people with limb difference in Uganda. This encouraged us to test a ‘limb buddy’ service, a phone-based, peer-to-peer support method used by the Koalaa team in the UK.

We recruited two Ugandan ‘limb buddies’ with trans-radial limb difference whose role was to offer peer-to-peer support to the other participants in the study. Both were male. LB1 (age 29) had congenital limb difference, 1 year of experience using a (non-Koalaa) prosthesis, and was fitted with a Koalaa just prior to the study. LB2 (age 21) lost his limb 1 year before the study as a result of trauma and had been fitted with a Koalaa ALX system 6 months before the study started. Each limb buddy provided informed consent, using either a signature or thumbprint.

### Koalaa ALX prosthesis

**Fig. 2:**
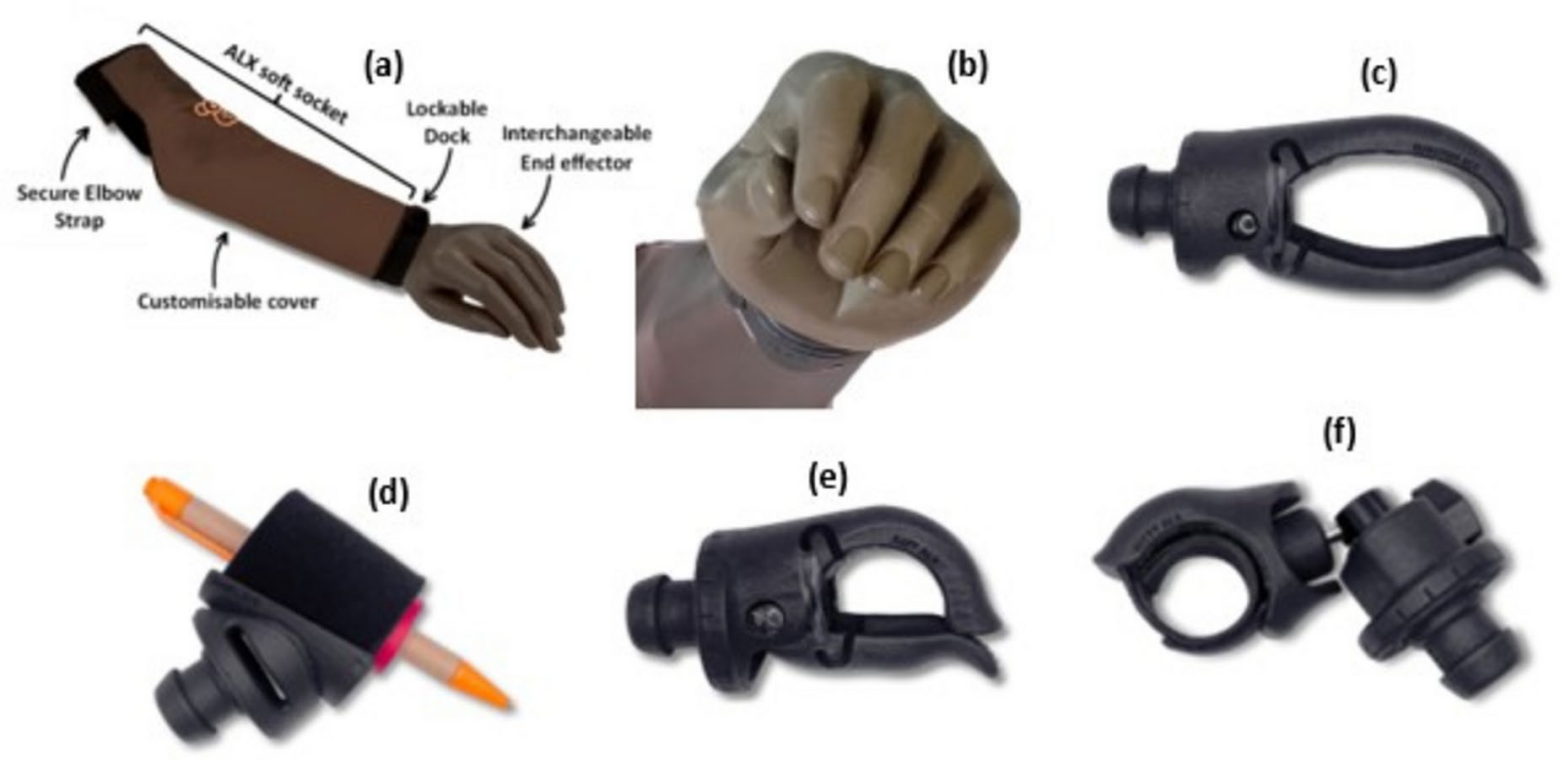
Koalaa ALX system. (**a**) The Koalaa ALX assembly; socket, dock, and end-effector, (**b**) Cosmetic hand, (**c**) Koalaa Rushton, (**d**) Koalaa Janet, (**e**) Koalaa Amy, (**f**) Koalaa Kitty.

The Koalaa ALX system is shown in Fig. 2a. The socket is cut to length, a lockable dock (wrist unit) is attached, and the user can choose which end effector to use at any given time. Participants and limb buddies were each supplied with an ALX socket, a cover, and a wrist unit. They were also provided with one (of a choice of two colours) cosmetic hand (Fig. 2b) and two functional voluntary-opening end-effectors, including the Rushton (Fig. 2c) and a choice between the Janet, Amy or Kitty (Fig. 2d-f) (https://www.yourkoalaa.com/alxtools*).* The orthopaedic technologists at FPRRH selected the two hand colours offered to participants.

### Protocol

**Fig. 3:**
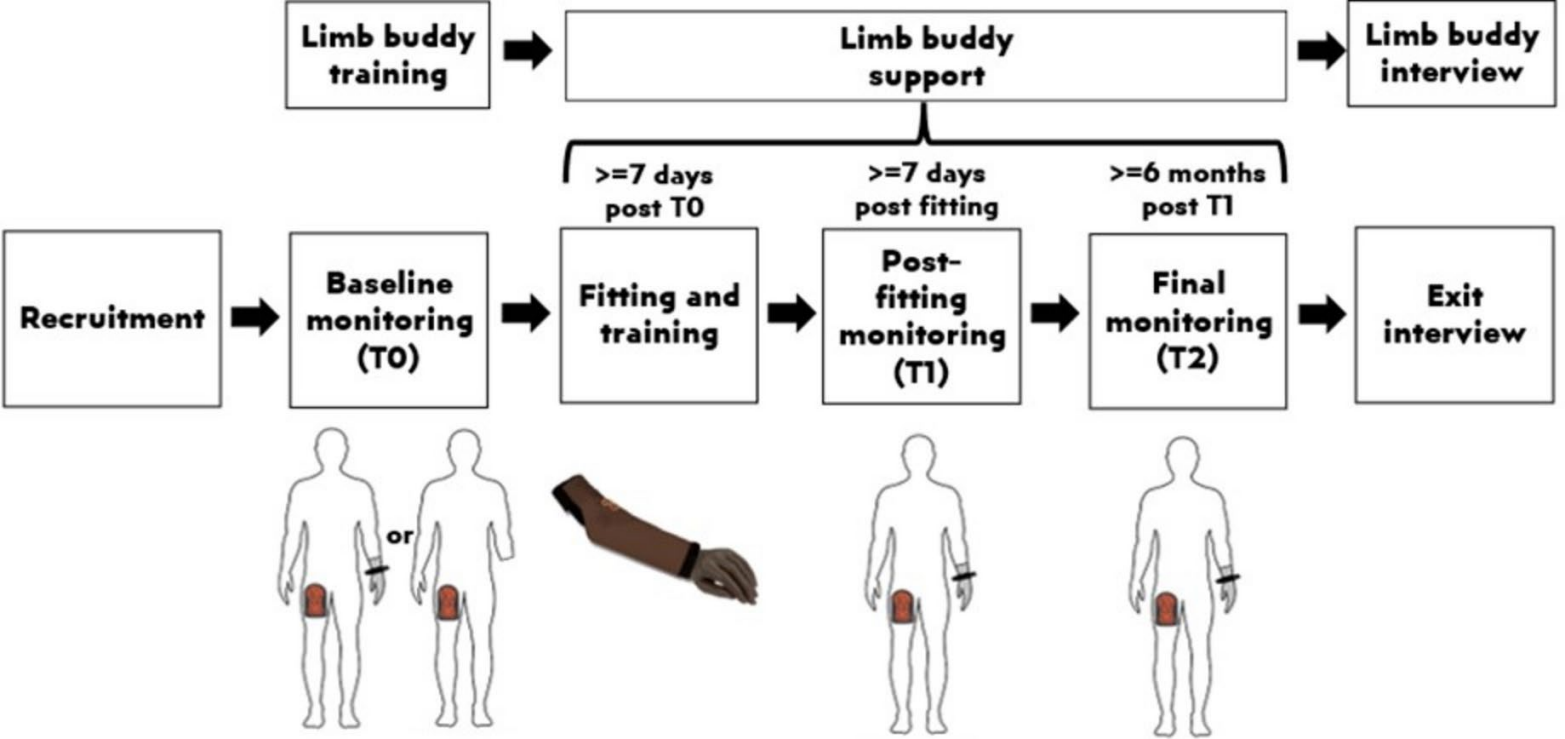
Study protocol.

The study protocol is shown in Fig. 3. In brief, to test the feasibility of capturing objective data on community participation, participants’ physical behaviours were recorded. To assess the value and acceptability of the ALX to users, prosthesis wear patterns were recorded using a wrist-worn sensor. Interviews were conducted with both the participants and limb buddies to record their experiences in the study.

### Limb buddies

Working with Koalaa and the local orthopaedic technicians, we developed a simple support package for the limb buddies. After obtaining informed consent, the two limb buddies were fitted with a Koalaa ALX system and provided with the same set of hands/end effectors as the patient participants (see Fig. 2). They were trained on how to use the device and given at least one month to accommodate to the new device. At the start of the study, we met with LB1 and LB2 at FPRRH workshop and informed them of the expectations of the research team and their role as limb buddies. After the training, we checked to see if they were happy to continue. The limb buddies were periodically provided with phone credit to contact the participants.

Over the course of the study, the two limb buddies were each allocated four of the eight patient participants and instructed to contact their participants three days and three weeks post-fitting, as well as monthly, for the length of their involvement within the study. When contacting each participant, the limb buddy would enquire about how the participant was adapting to the ALX, discuss any concerns or issues they may have regarding their prosthesis, provide advice on use and repairs, or suggest they visit the Fort Portal workshop when necessary. The participants were also encouraged to contact the limb buddy themselves if they needed advice or a repair. The contact information of the limb buddies was given to the patient participants at the fitting stage.

At the end of the study, both limb buddies were interviewed to capture their experiences. The interviews were adapted from the interview schedule for the patient participants (Online Appendix 1).

### Patient participants

#### Monitoring the use of the prosthesis and physical behaviours

To identify how the provision of the prosthesis and support/maintenance package impacts the users, we captured their real-world behaviours and prosthesis use. We used our previously developed method for tracking the patterns of wear and non-wear of upper limb prostheses from an Axivity 3-axis accelerometer (Axivity, Newcastle UK) mounted in a silicone Axivity wristband at the prosthetic wrist^16,17^. The Axivity monitors were initialised using the OMGUI Configuration and Analysis Tool software (v. 1.0.0.43, Axivity Ltd, Newcastle, UK) to record data at 50 Hz with a sensitivity of 16 g for at least 7 days.

To complement this wear data and inform on activity levels, we asked participants to wear an activPAL 4 + monitor (Pal Technologies, Glasgow, Scotland) on their thigh, which was attached using Hypafix adhesive dressing. ActivPAL and other similar monitoring devices have been used before to study the behaviour of lower limb prosthesis users^18^, but to the authors’ knowledge, they have not been used for upper limb prosthesis users. The activPAL 4 + includes a 3-axis accelerometer and a 3-axis magnetometer. The activPAL monitors were programmed to record data at 20 Hz. The resulting data were processed using the PALanalysis software (version 8.11.8.75) to indicate whether each minute epoch corresponded to sedentary activity, standing, stepping, cycling, primary lying, secondary lying, seated transport, or monitor non-wear. Secondary analysis of the activPAL data allowed for classifying activity into ‘at home’ or ‘community’ based on stepping characteristics. Participants were asked to keep an activity diary over the days they wore the activPAL monitors to assess the accuracy of the home vs community classification. The two monitors were programmed using the same computer, and synchronisation between the internal clocks of both sets of monitors was assessed (Online Appendix 2).

As shown in Fig. 3, there were three monitoring phases (T0, T1, and T2):

**T0 (baseline):** Each participant was asked to visit the orthopaedic workshop at FPRRH. Following informed consent, each participant was fitted with an activPAL monitor and instructed on its use. In the cases where participants entered the study owning a prosthesis, we took a photograph of the device, fitted an Axivity monitor on their prosthesis, and gave them instructions on its use. They were advised to keep the monitors on for at least 7 days. Participants then returned home. We covered the travel expenses of the participants at all phases.

**Fitting of the prosthesis:** The participant visited the workshop at a mutually agreeable date, at least 7 days after T0. The data from the monitor(s) was downloaded. The prosthetists fitted each participant with the Koalaa ALX system. While at the workshop, the researchers provided training on how to use the device and indications of the kinds of activities each end-effector may be best suited to before asking the participant to select their preferred end-effectors. In some cases, the participants tried out some activities using available tools at the workshop. The researchers also discussed possible repair scenarios with the participants and provided some spares and repair tools, including screws, rubber bands, pen holders (in the case of Kitty ALX), a screwdriver, and scissors. Participants then returned home to acclimatise themselves with the devices for at least 7 days. From this point on, the participants started receiving the limb buddy support package. Should the participant not wish to receive any further contact from either the limb buddy or the study as a whole, they were able to opt-out by telling the researcher.

**T1 (post-fitting data collection):** At least one week after fitting, participants from Kabarole district were invited once more to the FPRRH workshop, fitted with the activPAL monitor on the thigh and the Axivity monitor on the prosthetic wrist and asked to keep the monitors on for at least 7 days. We made arrangements to meet the participants from other areas at a mutually agreeable location. In some cases, for instance, when the participant had a shorter residual limb or concerns about wearing the Axivity monitor on their prosthetic wrist, it was embedded within the distal end of the ALX socket (proximal to the wrist unit). At least one week later, a research team member met with the participant to retrieve the monitors and download the data.

**T2 (monitoring 6 months following fitting and interview):** At least 6 months post-fitting, participants were contacted to arrange a meeting with the research team and undertake another activity monitoring period. All participants were asked to wear the activPAL on their thigh. They were asked whether they still used their Koalaa ALX. If they did, they were invited to wear the Axivity on the wrist of the ALX. If they came to the meeting without the Koalaa but instead with their original prosthesis, we invited them to wear the Axivity monitor on this device. If they self-reported having stopped using their prosthesis, we asked them to wear only the activPAL device on their thigh. At least 7 days later, we visited them once more to collect the monitors.

### Diaries

At T2, we also provided all participants who consented with a diary, which we asked them to fill out each time they either left or returned home over the 7-day monitoring period. For validation purposes, these data were compared with the results of the at-home/community classifier currently being developed on an able-bodied population in the UK, to check if the algorithm performed similarly in the Ugandan context^19^. We collected the completed diaries when we collected the monitors at the end of T2.

### Interviews

Irrespective of whether or not they stopped using their prosthesis, after the T2 monitoring period, we invited the participants to take part in a one-on-one semi-structured interview on their experiences, which lasted approximately 20 to 45 min. The interview guide is available in Online Appendix 1. One of the Ugandan research team members conducted the interviews in English, Luganda, or Rutooro, depending on the interviewee’s preference. The same researchers carried out transcription and translation into English.

### Analysis

#### Prosthesis wear periods

Data were downloaded from the Axivity sensors using the OMGUI software, resampled at 50Hz, and then exported as *.wav* format. To allow for the use of previously published analysis techniques developed for Actigraph sensors (Actigraph LLC, Pensacola, FL, USA)^16,17^, the data collected using the Axivity AX3 sensors was converted into values equivalent to ActiGraph activity counts using the method reported by Brønd et al.^20^.

Data were then grouped into 60-s epochs, and the activity counts across the three axes were combined into a resultant vector magnitude value. The 60s epoch vector magnitude data for sensors were passed through a non-wear algorithm developed by Chadwell et al.^16^ to identify periods when the sensors were not worn (https://github.com/AlixChadwell/ProsthesisNonWearChadwell).

As prosthesis users were instructed to keep the sensor on the wrist of the prosthesis throughout the study, regardless of whether the prosthesis was worn at the time, it was assumed that non-wear of the prosthesis-worn sensor would constitute non-wear of the prosthesis. As the sensor was wrist-mounted, the data provides no indication of which (if any) of the prosthetic end effectors was being worn.

#### Physical behaviours

Data were downloaded and saved as *PAL.Datx* files from the activPAL4 + monitors using PALconnect, part of the PAL software suite (https://www.palt.com/softwaresuite/). PALanalysis and PALbatch software were then used to apply the built-in GHLA v2.2 algorithm (an updated version of the CREA algorithm for activPAL 4 + devices)^21^. This software was used to group the data into 60s epochs, with each 60-s epoch broken down into a summary number of seconds for a variety of different activity classifications. The resulting CSV file contained a breakdown of activity classifications and the number of steps determined by the algorithm for each minute epoch throughout the entire data collection period. The classifications included sedentary (sitting), upright (standing), cycling, primary lying (sleeping), secondary lying, seated transport, and non-wear. Each 60-s epoch was labelled according to the dominant activity within that period.

#### Home and community

ActivPal data were classified as occurring either ‘at home’ or ‘away from home’ (assumed to be in their community) based on transition events. A transition event was defined as a participant continuously stepping for more than 60 s or for more than 25 s in a straight line, a behaviour that is very unlikely to be observed within a constrained environment, such as a participant’s home^22^. By identifying the first time a transition event occurs after a period of sleep (leaving home), and the final time a transition occurs before a period of sleep (returning home), we can therefore establish a measure of community participation, by quantifying the active hours each day (from midnight to midnight). This was applied only on days where more than 20 h of data was collected from the activPAL monitor. The results provide insight into whether provision of the Koalaa ALX was associated with a change in community participation.

### Combining data on prosthesis wear and physical behaviours

To provide insight into the behaviours associated with prosthesis wear and non-wear, we report on the number of 60s epochs during which the participants were stepping, in each 30-min window pre- and post- each prosthesis donning and doffing event.

In Online Appendix 3 we present plots showing the synchronised wear and physical behaviour data, for each participant, as well as the duration of simultaneous recording periods for the ActivPal and Axivity monitors.

### Interviews

Analysis of the qualitative interviews followed the 6 phases set out by Braun and Clarke^23^. The data familiarisation phase was undertaken in the field, working alongside Ugandan co-researchers. This enabled us to check some of the specifics (for example, dates and facilities) and ask further questions about cases where necessary. Initial code generation took place in this co-researching context, initially on paper and then in a trial NVivo project (NVivo 12, QSR International).The process of achieving a shared understanding of initial themes generated inductively from the narratives inevitably leads to the overwhelming ‘messiness’ that necessarily characterises qualitative research^24–26^. The themes were then reviewed and redefined to shape a meaningful structure, a new NVIVO project was opened for coding, and writing-up commenced (led by co-author Ackers). Writing is an integral part of reflexive data analysis and reflections on the data led to continuous review of the themes and their relationships to each other.

## Results

Table 2 shows a summary of the choices of ALX tools by participants at fitting and whether they were still using the Koalaa ALX device up until T2 monitoring period. P3 and P8 wore their original prosthesis for T2 monitoring.

**Table 2:** A summary of ALX tool selection and device usage at T2. All participants were provided with one cosmetic hand and a Rushton ALX. * Neither P3 nor P8 had access to their Koalaa ALX device during T2.

Participant number	Choice of ALX tool			Using ALX socket at T2
	Janet	Amy	Kitty	
P1	☑			Opted out at T2
P2		☑		Y
P3			☑	*N
P4		☑		Y
P5		☑		Y
P6			☑	Y
P7			☑	Y
P8			☑	*N

**Fig. 4:**
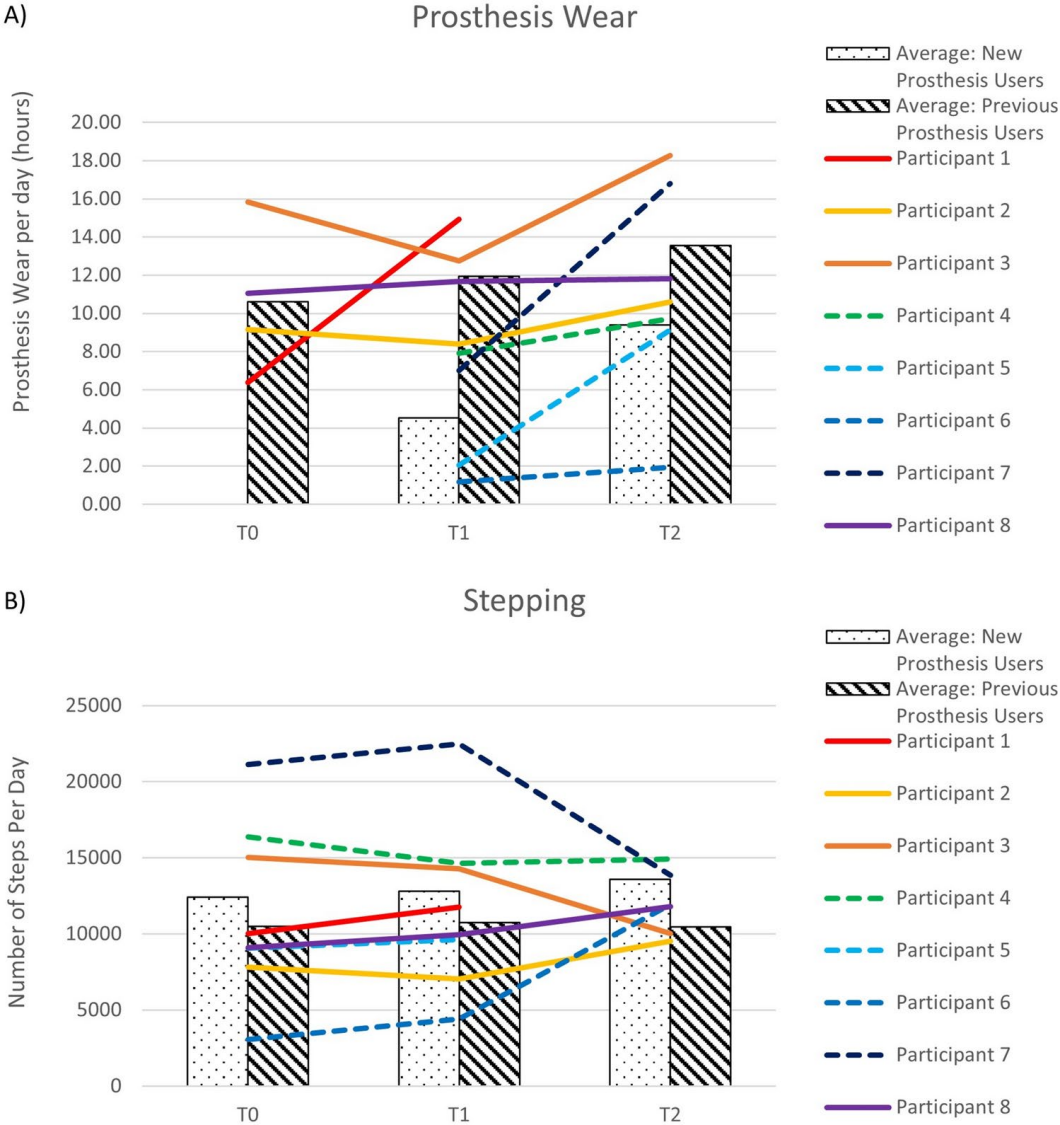
(**a**): Prosthesis wear time (in hours/day) for participants who entered the study with a prosthesis (Previous prosthesis users- Participants 1,2,3,8) and participants with no prior experience of prosthesis use (New prosthesis users – Participants 4,5,6,7). Note that P3 and P8 used their previous prosthesis (not the Koalaa) at T2. Figure 4 (**b**): Number of steps/day for participants who entered the study with a prosthesis (Previous prosthesis users- P1,P2,P3,P8) and participants with no prior experience of prosthesis use (New prosthesis users – P4,P5,P6,P7). In all cases new users are indicated by dashed lines. Note that P3 and P8 used their previous prosthesis (not the Koalaa) at T2.

Figure 4a shows that participants who entered the study with a prosthesis wore them for an average of 10.61 h per day. The average wear time recorded shortly after fitting with a Koalaa was slightly higher (T1). For the three participants who continued in the study, wear time/day reached 13.56 h at T2. P3 and P8 were wearing their original prosthesis when they started the T2 monitoring period and hence wear times for these participants at T2 reflects wear of their original devices. P8 used his original prosthesis for (his manual) work and his T2 data collection coincided with an extended work period away from home (he reported predominantly using his Koalaa at home). P3 lost access to his Koalaa between T1 and T2 due to circumstances external to this study. The ActivPal monitor for P5 at T2 stopped recording after less than 24 h, so the data are excluded.

The participants who had no prior experience of wearing a prosthesis showed wear time of 4.53 h/day at T1. For each of the participants, wear time increased, in some cases substantially, at T2, to an average of 9.4 h/day.

Steps/day for the participants who entered the study with a prosthesis remained between 10 and 11 thousand over the course of the study. For participants for whom the Koalaa was their first experience of using a prosthesis, steps/day increased from 12,406 at baseline to 14,647 at T2 (Fig. 4b).

**Fig. 5:**
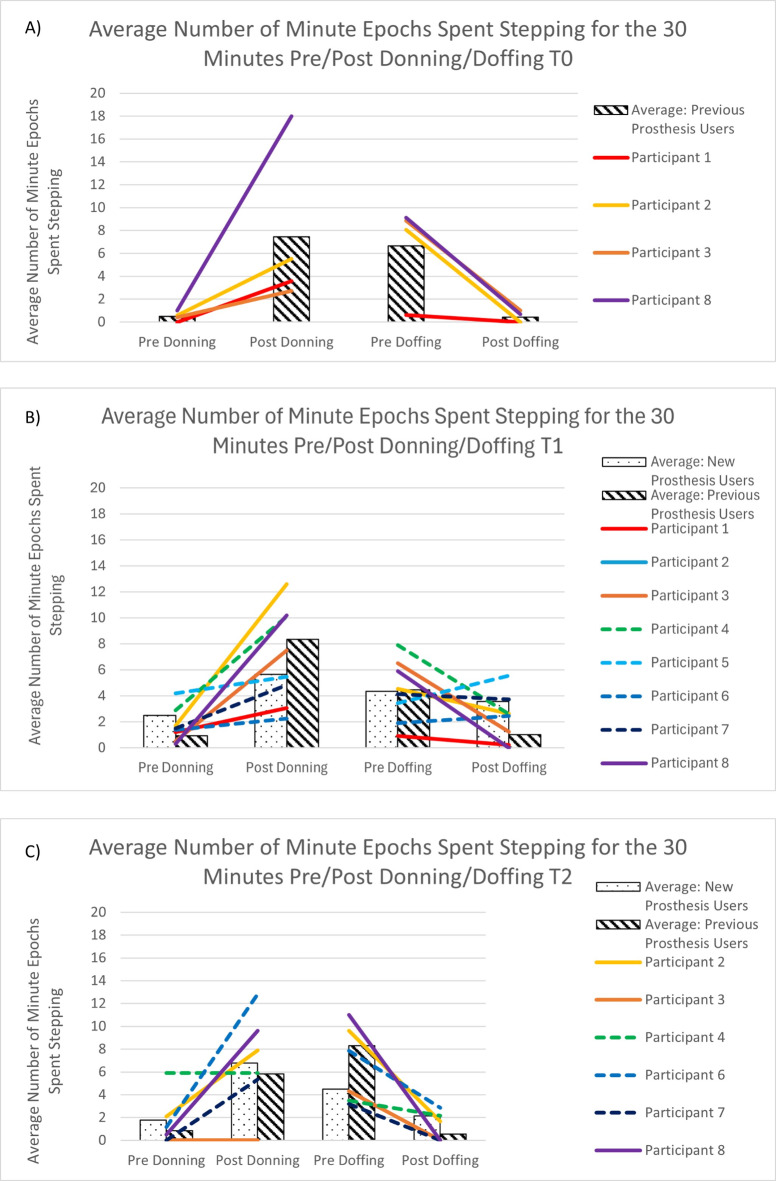
(**a**): Average number of minutes stepping for the 30 min pre- and post-donning and doffing events (T0) for participants who entered the study with a prosthesis (Previous prosthesis users- P1,P2,P3,P8). Figure 5 (**b**): Average number of minutes stepping for the 30 min pre- and post-donning and doffing events (T1) for participants who entered the study with a prosthesis (Previous prosthesis users- P1,P2,P3,P8) and participants with no prior experience of prosthesis use (New prosthesis users – P4,P5,P6,P7). Figure 5 (**c**): Average number of minutes stepping for the 30 min pre- and post-donning and doffing events (T2) for participants who entered the study with a prosthesis (Previous prosthesis users- P1,P2,P3,P8) and participants with no prior experience of prosthesis use (New prosthesis users – P4,P5,P6,P7). Note that P3 and P8 used their previous prosthesis (not the Koalaa) at T2 and P5 is not shown due to the short length of the recorded data (Supplementary Table 2).

Participants who entered the study with their own prosthesis showed increased stepping activity in the 30-min periods post-donning, and pre-doffing (i.e. immediately after putting the prosthesis on, and immediately before taking it off) at T0. Stepping was rarely observed in the periods immediately after taking the prosthesis off, or prior to putting it on (Fig. 5a).

We see a similar pattern of stepping either side of donning or doffing events in the previous prosthesis users at T1, and a somewhat similar, but less distinct, pattern amongst the participants for whom the Koalaa was their first prosthesis (Fig. 5b).

By T2 (6 months post-Koalaa fitting), both groups showed increased stepping immediately after donning and immediately before doffing their prostheses, with reduced stepping evident immediately post doffing their prosthesis (Fig. 5c).

**Fig. 6:**
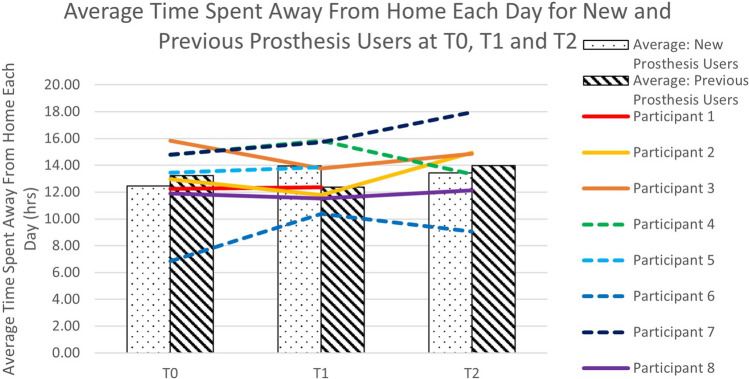
Average time spent away from home each day for participants who entered the study with a prosthesis (Previous prosthesis users- P1,P2,P3,P8) and participants with no prior experience of prosthesis use (New prosthesis users – P4,P5,P6,P7). Note that P3 and P8 used their previous prosthesis (not the Koalaa) at T2.

The time spent away from home was similar across both groups and across the three time points (T0,T1, T2), with a small increase noted at T1 and T2 for the three new prosthesis users for whom we had sufficient data (Fig. 6).

Seven of the eight participants and both limb buddies agreed to the exit interview. Between T1 and T2, P1 stated he no longer wanted to be involved with the study.

Several themes emerged from the interviews related to their experiences of the study itself and to the Koalaa ALX. These are discussed below.

### Experience of wearing the Koalaa ALX sleeve

Respondents were generally satisfied with the experience of wearing their Koalaa prosthesis. For instance, P5 stated, “*I have not found any difficulties because I can do everything on my own, including wearing it [tightening it], removing it, I can*!” P8 preferred the appearance of the Koalaa prosthesis to his previous (metal) hand and said, “*Before I got the [Koalaa device], I am not very proud of this one with the metallic hand, especially when I have gone in public; it is suitable for only my work. But with the Koalaa, I go with it in public, and I feel that I fit in well.*” P2 also favourably compared the Koalaa with her previous prosthesis: “*The socket, it is very fine, it is not heavy. By the way, I had forgotten to tell you the other one was too heavy, this one is not heavy, and there is no heat*”.

However, some mentioned discomfort and approaches to addressing this. For instance, P8 said, “*Maybe when you have just started using it, the [rivet] at the elbow joint [painfully presses against your skin] when you are carrying something so, at many times when I was wearing the device, I would wear it with like two sock*s” Others reported unpleasant sensations when they first started wearing the device, but that these went away with time “*At first, it was heavy, because my stump was still painful. But after getting used to putting it on, I have not had any issues with it*” (P4), and P6 stated, “*So the problem I found is that I was slow to get used to it. But once I got used to it, I have no problems now. It was heavy at first*.”

Patterns of activity while wearing different end effectors came out in the interviews. P3 stated, “*[Rushton ALX] But it helped me all the same to wash clothes, when I was going to the well, I would hold an empty twenty-litre jerrycan and a three-litre jerrycan with water in it, I would be able to carry it*.” P7 commented, “*If I need to ride my bicycle, I take this [cosmetic hand] off, and I put on the [Rushton ALX], and it holds the bicycle, so I can ride away. Even when I am bathing, I put on the [Rushton ALX], I put a sponge into it, and I can start scrubbing [my body]*.” Several participants commented positively on the wrist unit, which locked the hand in position and allowed for straightforward swapping of end effectors.

P5 describes how, when she first received the Koalaa device, she was afraid of using it and people seeing it, as her family and the wider public had not been exposed to this type of technology. Reflecting the patterns seen in Fig. 5, she reported not using the device much for the first 3 months until her parents encouraged her to try it out: “*When I had just received it, I was afraid of it. I would put it on and even fear to leave the house. But eventually, when [my parents] advised me, I put in on, and now I am free with it.”* After using it for several months, her outlook changed: “*Now, when I put it on and walk [about in public], I do not feel any shame from people. Apart from those making fun of me [in a joking manner], but personally, I do not have any problem with the device. I no longer feel afraid that people will see me and get scared.*”

P2 stated, *“When I’m at home, I don’t [wear it] because I have to do some activities, but when I’m outside, I do, even when I’m in classes I do, because I use a right hand to write, whereby this one [referring to Koalaa device] is a left hand side. Wherever I’m outside, I’m always putting it on, apart from home, at home I do a lot of work and whereby I have to remove it, and I do my work without it because there’s no way it can support me when I’m cleaning the house.”*

### Activity monitoring

We asked participants to comment on their experiences of wearing the two monitors. Initial concerns of one participant regarding wearing an electrical device wore off over time: “*I was a bit worried when you mentioned that the device is rechargeable. [I wondered whether it would affect me in any way, given that I have been told to have excess electricity in my body]. While I was worried, I took my mind off it slowly*” (P5). Other people’s (particularly partner’s) initial reactions to the monitors were also mentioned by several participants. Initial responses included fear “*the first time we got them, [my wife] was scared*” (P4) and “*my wife is suspicious because the monitors [activPal] keep blinking*” (P3). Marks on the skin resulting from the activPal were mentioned by two participants, and itching was reported by several.

The Axivity monitor, worn on the prosthetic wrist, was generally well accepted. Several participants commented that other people often mistook the monitor for a watch and that this was, in some cases, seen as a positive; for example, “*They could think it was a watch and even I could brag about it*” (P2).

### Cleaning

The interviews suggest that respondents didn’t get clear advice at the time of fitting on how to clean their Koalaa sleeve, and most respondents worried about how to clean it and prevent any damage. Several spoke of simply wiping the exterior of the device, but most respondents wanted to wash the inner layer, which absorbed sweat. Respondents reported washing and, in two cases, ‘scrubbed’ the device using detergent. Two respondents had come up with their own ‘solutions’ choosing to use a sock insert to try to keep the device clean and also mitigate sweating: *“When I am wearing the device, I put on socks, so it does not touch my skin. When I am cleaning, I only clean the socks, I do not touch the device (P8)”.* This also reflected a wider concern about the plastic material used for the cover, which contributed to sweating, especially in a warm environment.

While much of the discussion about stigma focuses on the aesthetic appearance of a device, the smell from the device impacted use in some cases; “*And even there’s some scent inside [*the socket*], which I’m not comfortable with. I have to wash it” (P2).* A limb buddy described how it took a whole day for the device to dry out properly when he washed it. One respondent asked if it may be possible to have more than one device or perhaps additional cover(s) so they could wash it and leave it to dry out while using a second device.

### Repairs

**Fig. 7:**
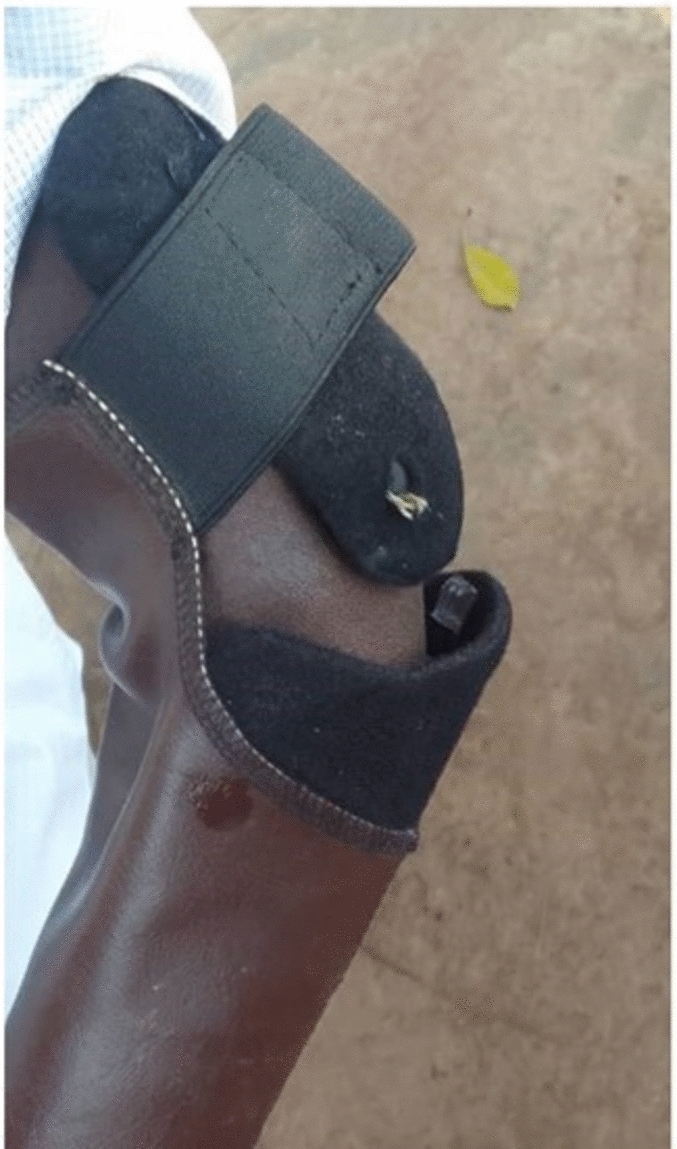
Example of failure of a rivet.

Four respondents said they required some form of repair. The observed weaknesses in the device include the elastic bands used in the Amy and Rushton grippers (Fig. 2), which frequently break, especially during heavy use. Other failure points include rivets used to attach the sleeve to the above-elbow component (Fig. 7), Velcro fasteners for the cover (referred to by some interviewees as ‘the carpet’), and, over the longer term, wearing out of the sleeve component. As respondents got used to the device and began to identify it as an integral part of their body, they became anxious about repairs and access, especially to these components, to the extent that they may try to ration their own use to safeguard the device. P8, for instance, who has a welding job, stated, “*For my work, when I am welding, I use my metallic hand to hold the [large welding cable]. I think if I were to use the [Koalaa device], the fingers might break off. So, I use it when I am going somewhere where I am not going to do any heavy work*.”

Respondents viewed their participation in the ‘research’ project as a ‘gift’ but were concerned about what happens at the end of the process. Nearly all respondents expressed a ‘hope’ that they could contact the limb buddy or project staff after the end of the project should they face any problems. This was less of a concern for participants who lived near to and were familiar with FPRRH, as they said they would simply return. However, several respondents lived many miles away and spoke of trying to access regional workshops who had no familiarity with these devices.

Several respondents had either considered self-repair or made their own repairs. For example, P2 said, “*When my hand got separated, like the hinge joint, those buttons they got broken, but to me, because I have been going to many hospitals, I decided to work on it without asking permission. I joined it because I’m in love with my hand, I feel uncomfortable when I am not putting it on.”* In another case, the respondent had spent many years repairing his previous metal device (Fig. 1) and reflected the extent to which he assumed responsibility for it and, perhaps, didn’t expect to be able to access repair services; [P8] “*There are times that I have needed to repair my (old) device (he got from FPRRH). [With the metallic hand], there was a time one of the screws fell out, and I replaced it with a tapping screw, and it held. There was another time when some of the [fingers] broke off, and I welded them back myself. If [the Koaala needs repair] and I can repair the damage myself, I would do it. If not, I would look for you who gave me the device because you know more about it.”* Another respondent (P4) described how he accessed a repair at a local cycle repair shop: “*When the [rivet at the elbow connector] got out, I called you and informed you about it. And because it [the device] is now part of my body, by the time you gave me the advice [to go to a local bicycle or shoe repair shop or tailor shop], I had already thought about it. I went to a [local] bicycle mechanic, and he repaired it”.*

### Limb buddies

Both limb buddies reported that they offered most of the support to the patient participants but were rarely called upon to answer ad-hoc questions. LB1 stated, “*Yeah, I think I often contacted them, but they did not contact me all that. Because I could remember and call them, ask them. Oh yeah, it’s only one person who contacted me when he got challenges with putting it on. And I also shared a video with him [showing him] how I do, how I put on mine*.” When LB1 called participants, “*most of the times, to those I call, I call them, I greet them and they ask me. I don’t think, because by the time I call them, it’s not because they have a challenge with the hand. I call, and then we interact. I ask them whether they have challenges, they say no, everything is okay.*” LB2 reported a similar experience: “*I got one person for a limb buddy, and he was asking me in terms of washing, so I was ready to answer him. And he was also asking me in terms of repair. I also answered him*.”

Patient participant interviews generally corroborated the picture that emerged from the LB interviews. For example, P3 stated, “*In the beginning, I got an issue where I was overly tightening my socket, but when I asked him [Limb Buddy], he made me a video and showed me how to properly tie my socket. When I did it this way, I did not get any more issues with my socket”.* P4 reported that encouragement from the LB to overcome initial discomfort associated with wearing the Koalaa encouraged long-term use: “*At the start, when I felt the device was heavy, when I was in pain, I called him, and he encouraged me that I would get used [to using the device], and finally, I got used to it. Every time something changed, I called him to give an update*”. Others reported little interaction with their LB (P6, P7, P8).

## Discussion

Our study has shown how the Koalaa, an off-the-shelf adjustable prosthetic system, together with a small support package, can be deployed in a Ugandan public healthcare setting. It is not uncommon for researchers who develop new upper limb prosthetic technologies to make claims of suitability for low resource settings, based typically on unit cost or the potential for local production through 3D printing or similar techniques^27^. However, very few studies evaluate the deployment of such devices in a low-resource setting (e.g.^28^), and to the best of our knowledge, no previous study has reported objective data on the real-world use of upper limb prostheses in such settings.

The Koalaa system’s off-the-shelf adjustable socket bypasses one of the key bottlenecks to the much-needed scaling up of prosthetic services, the complex processes involved in traditional socket manufacture. Fitting took around 20 min, which is likely to be one or more orders of magnitude faster than the time taken to produce a conventional socket. It also has a wrist unit, an appropriately coloured cosmetic hand, and some simple grippers. The combination is demonstrably acceptable to our participants, at least over the short term. Consistently high rates of rejection of both powered and non-powered prosthetic devices have been reported in well-resourced settings^29,30^, but to the best of our knowledge, there are no studies reporting on rejection rates in users of upper limb prostheses living in low-resource settings.

The four participants who entered the study with a prosthesis wore their original prosthesis for an average of over 10 h/day at T0. Similar wear times were seen when first provided with the Koalaa (T1), and average wear time reached over 13 h/day at T2 (Fig. 4a). A small number of studies have reported objective data on wear times in populations of upper limb prosthesis users who live in high resource settings. Chadwell et al.^16^ found a median wear time of 6.5 h/day in her UK-based study of 20 trans-radial myoelectric prosthesis users, with only 5 of her participants wearing the prosthesis for > 13 h per day. Frey et al. reported average wear time of 11.1 h/day in a US-based population of 22 users of a variety of upper limb prostheses. Interestingly, although our participants, and others in previous work, have expressed a desire for the colour of prosthetic hands (or gloves) to match their skin tone and/or to closely resemble the appearance of an anatomic hand, three out of the four participants’ hands did not meet one or both of these criteria (Fig. 1). The participants who were new to using a prosthesis showed relatively infrequent wear of the Koalaa at T1, but wear time increased to a level that was similar to that seen in Chadwell’s study by T2. Occupational Therapy input to build confidence and skill in upper limb prosthesis use is not available in Uganda, but P5’s interview suggested that encouragement from her parents may have offered her similar support. This may be reflected in the increase in wear time from ~ 2 (T1) to ~ 9 h at T2 (Fig. 4a). Future studies in low resource settings could explore the role of informal support networks further.

Our study appears to be the first to combine real-world monitoring of upper limb prosthesis wear patterns with objective data on user behaviours. We know from other work that upper limb amputees report problems with participation following limb loss^9^, including work and social events. Our earlier work in Uganda found that people reported restricting their activities outside of the home, in part due to the stigma associated with ‘undisguised’ limb difference^31^. We observed a clear increase in stepping immediately post-donning and a decrease immediately post-doffing the Koalaa prosthesis in both new and previous prosthesis users by T2, suggesting there may be a relationship between prosthesis wear and activity levels. P2 mentions that he always puts the prosthesis on when outside, and takes it off when he returns home, a pattern that is reflected in his T2 data (Fig. 5c).

However, we did not see a very clear pattern of increase in the overall number of steps/day across all participants who were new to a prosthesis, which might be expected based on our previous work. Average time away from home increased slightly by T2. However, both of these observations were partly confounded by limited activPAL datasets at T2 for (particularly) P5 and P6. One of the activPAL monitors was found to be faulty, and in other cases, the monitors were not fully charged before deployment. We didn’t probe any changes to employment across individuals, and future studies should address this.

Repairs, ongoing and long-term support were both issues that participants commented on in detail. The Limb Buddy system appeared to work satisfactorily, and some participants clearly benefited from it. Informal networks of people with upper limb difference exist in Uganda, and it may be that these networks offer an informal version of the ‘limb buddy’ system. However, the variety of devices used in Uganda means that knowledge of any particular type of prosthesis may be difficult to access via an informal network. The overhead to implementing the Limb Buddy system was relatively small, and further work to investigate this model is warranted. Clearly, there were some design issues with the Koalaa that need to be addressed (e.g., Fig. 7), and Koalaa has since re-designed the connection at the elbow joint. Another very common failure was the snapping of the rubber bands used in the Rushton and Amy grippers. Participants were given multiple spares at first fitting, but the frequency with which these were snapping meant that, in some cases, they ran out and sought support from other centres. Audits show that users of prostheses unsurprisingly experience regular need for repairs^32,33^, and a better understanding of the relative robustness of different prosthetic devices would help inform future prosthetic prescription decisions.

Consistent with our previous study^8^, participants accessed repairs via various routes, including orthopaedic workshops, other types of workshops (e.g., bicycle repair workshops and tailor shops), and self-repair. Understandable concerns were raised regarding the long-term support for users beyond the end of the study. Ethical guidelines^34^ on conducting research in low resource settings were followed; participants who entered the study with their prosthesis were advised that they could keep their own device, and all participants were allowed to keep the Koalaa after the end of the study. We also kept a stock of spare parts at the FPRRH for use by participants beyond the end of the study. However, the longer-term support for these participants remains a challenge, which we are actively seeking solutions to. Our partner, Knowledge for Change, has recently established itself as a distributor of prosthetic and orthotic materials and components, and discussions with Koalaa are ongoing. In addition, some of our team are contributing to the first Ugandan National Rehabilitation plan, which may lead to government funding for services and hence some capacity to generate public sector demand for devices such as the Koalaa. The challenges of sustaining and scaling up the provision of devices, such as the Koalaa in sub-Saharan African countries is discussed in more detail in^35^.

Thermal discomfort in sockets is a well-known issue^36^, and others have reported concerns over unpleasant smells associated with sweat build-up in sockets^37^. Although several respondents reported no issues when asked explicitly about thermal discomfort, others reported some discomfort. Our findings suggest that advice on cleaning the socket is an important issue that should be raised with participants.

## Conclusions

Our study has shown that a novel upper limb prosthesis, the Koalaa ALX, can be successfully deployed in a public sector service in Uganda and appears to be acceptable to both users of existing devices, and to people with no prior experience of prosthesis use. Our novel methods for capturing objective data on real-world use of the devices were acceptable to users and provided new insights into the relationships between wear and physical behaviours. Repairs and long-term support for devices are areas for further attention.

## Supplementary Information

Supplementary Information.

## Data Availability

The ActivPal and Axivity datasets are available from the University of Salford Figshare at [10.17866/rd.salford.28173860.v1].

## References

[1] Yuan, B., Hu, D., Gu, S., Xiao, S. & Song, F. The global burden of traumatic amputation in 204 countries and territories. *Front Public Health* **11**, 1258853. 10.3389/fpubh.2023.1258853 (2023). 37927851 10.3389/fpubh.2023.1258853 PMC10622756

[2] 2World Health Organization & United Nations Children’s Fund (UNICEF). Global report on assistive technology. World Health Organization, Geneva, (2022).

[3] International Diabetes Federation. In: *IDF Diabetes Atlas* International Diabetes Federation, Brussels, Belgium, (2021).

[4] Mulindwa, B. et al. Evaluation of the current status of prosthetic rehabilitation services for major limb loss: a descriptive study in Ugandan Referral hospitals. *Disabil. Rehabil.*10.1080/09638288.2023.2188266 (2023). 36960619 10.1080/09638288.2023.2188266

[5] Kenney, L. *et al.* in *Global perspectives on assistive technology: proceedings of the GReAT Consultation 2019* Vol. 2 414–426 (World Health Organisation, Geneva, 2019).

[6] Huck, J. J., Atim, P., Moro, E. B. & Nirmalan, M. Prevalence and Spatial Patterns of Major Limb Loss in the Acholi Sub-Region of Uganda. *Prosthesis* **4**, 369–382 (2022).

[7] Okello, T. et al. Major limb loss (MLL): an overview of etiology, outcomes, experiences and challenges faced by amputees and service providers in the postconflict period in Northern Uganda. *J. Glob. Health Rep.*10.29392/joghr.3.e2019028 (2019).

[8] Oldfrey, B. et al. Repair strategies for assistive technology in low resource settings. *Disabil. Rehabil.*10.1080/17483107.2023.2236142 (2023). 37466362 10.1080/17483107.2023.2236142

[9] Hutchison, A., D’Cruz, K., Ross, P. & Anderson, S. Exploring the barriers and facilitators to community reintegration for adults following traumatic upper limb amputation: a mixed methods systematic review. *Disabil. Rehabil.*10.1080/09638288.2023.2200038 (2023). 37042419 10.1080/09638288.2023.2200038

[10] Morgado Ramirez, D. Z. et al. The lived experience of people with upper limb absence living in Uganda: A qualitative study. *Afr. J. Disabil.*10.4102/ajod.v11i0.890 (2022). 35747758 10.4102/ajod.v11i0.890 PMC9210140

[11] AT2030. Product narrative: Prostheses. A market landscape and strategic approach to increasing access to prosthetic devices and related services in low- and middle-income countries. (2020).

[12] Baldock, M. et al. Adjustable prosthetic sockets: a systematic review of industrial and research design characteristics and their justifications. *J. Neuroeng. Rehabil.*10.1186/s12984-023-01270-0 (2023). 37926807 10.1186/s12984-023-01270-0 PMC10626671

[13] Smail, L. C., Neal, C., Wilkins, C. & Packham, T. L. Comfort and function remain key factors in upper limb prosthetic abandonment: findings of a scoping review. *Disabil. Rehabil. Assist. Technol.* **16**, 821–830. 10.1080/17483107.2020.1738567 (2021). 32189537 10.1080/17483107.2020.1738567

[14] Ackers, H. *Patient Satisfaction with Services at FPRRH’s Orthopaedic Workshop 2021–2024* (2024).

[15] Yagos, W. O., Olok, G. T., Moro, E. B., Huck, J. & Nirmalan, M. Use of mobile phones for rehabilitative services among prosthetics users in rural Acholi sub-region of northern Uganda: findings from a qualitative study. *BMC Med. Inform. Decis Mak.* **22**, 263. 10.1186/s12911-022-02008-z (2022). 36207722 10.1186/s12911-022-02008-z PMC9547425

[16] Chadwell, A. et al. Upper limb activity in myoelectric prosthesis users is biased towards the intact limb and appears unrelated to goal-directed task performance. *Sci. Rep.* **8**, 11084. 10.1038/s41598-018-29503-6 (2018). 30038402 10.1038/s41598-018-29503-6 PMC6056489

[17] Chadwell, A. et al. Visualisation of upper limb activity using spirals: A new approach to the assessment of daily prosthesis usage. *Prosthet. Orthot. Int.* **42**, 37–44. 10.1177/0309364617706751 (2018). 28650213 10.1177/0309364617706751 PMC5808815

[18] Chadwell, A. et al. Technology for monitoring everyday prosthesis use: a systematic review. *J. Neuroeng. Rehabil.* **17**, 93. 10.1186/s12984-020-00711-4 (2020). 32665020 10.1186/s12984-020-00711-4 PMC7362458

[19] Gracey-Mcminn, L. *et al.* in *The 8th International Conference on Ambulatory Monitoring of Physical Activity and Movement (ICAMPAM 2024).*

[20] Brond, J. C., Andersen, L. B. & Arvidsson, D. Generating ActiGraph Counts from Raw Acceleration Recorded by an Alternative Monitor. *Med. Sci. Sports Exerc.* **49**, 2351–2360. 10.1249/MSS.0000000000001344 (2017). 28604558 10.1249/MSS.0000000000001344

[21] Buchan, D. S. & Ugbolue, U. C. Comparing the activPAL CREA and GHLA Algorithms for the Classification of Postures and Activity in Free-Living Children. *Int J Environ Res. Public Health*10.3390/ijerph192315962 (2022). 36498039 10.3390/ijerph192315962 PMC9739422

[22] Speirs, C., Granat, M., Stamatakis, E. & Hamer, M. Estimating changes in physical behavior during lockdowns using accelerometry-based simulations in a large UK cohort. *Scand. J. Med. Sci. Sports* **31**, 2221–2229. 10.1111/sms.14032 (2021). 34378241 10.1111/sms.14032

[23] Braun, V. & Clarke, V. Using thematic analysis in psychology. *Qual. Res. Psychol.* **3**, 77–101. 10.1191/1478088706qp063oa (2006).

[24] Cook, T. The importance of mess in action research. *Educ. Action Res.* **6**, 93–109. 10.1080/09650799800200047 (1998).

[25] Cook, T. The purpose of mess in action research: building rigour though a messy turn. *Educ. Action Res.* **17**, 277–291. 10.1080/09650790902914241 (2009).

[26] Humble, D. This isn’t getting easier”: Valuing emotion in development research. *Emot. Space Soc.* **5**, 78–85 (2012).

[27] Ten Kate, J., Smit, G. & Breedveld, P. 3D-printed upper limb prostheses: a review. *Disabil. Rehabil.* **12**, 300–314. 10.1080/17483107.2016.1253117 (2017). 10.1080/17483107.2016.1253117 28152642

[28] Lopez, J. S. *Accessible Hand Prostheses: 3D Printing meet Smartphones* PhD thesis, Technical University of Delft, (2021).

[29] Magnus, P. et al. Prosthesis rejection in acquired major upper-limb amputees: a population-based survey. *Disabil. Rehabil.* **7**, 294–303. 10.3109/17483107.2011.635405 (2012). 10.3109/17483107.2011.635405 22112174

[30] Salminger, S. et al. Current rates of prosthetic usage in upper-limb amputees - have innovations had an impact on device acceptance?. *Disabil. Rehabil.*10.1080/09638288.2020.1866684 (2020). 33377803 10.1080/09638288.2020.1866684

[31] Morgado Ramirez, D. Z. *et al.* The lived experience of people with upper limb absence living in Uganda: A qualitative study. *2022* **11**, 10.4102/ajod.v11i0.890 (2022). 10.4102/ajod.v11i0.890 PMC9210140 35747758

[32] Berthaume, M. et al. Demographic, medical, and financial statistics from the Jaffna Jaipur Centre for Disability Rehabilitation (JJCDR) database, 1987–2018: a prosthetics, orthotics, and mobility clinic in northern Sri Lanka. *J. Global Health Rep.*10.29392/001c.88105 (2023).

[33] H. Nagaraja, V., Cheng, R., Henderson Slater, D., Thompson, M. & Bergmann, J. 2022 Upper-Limb Prosthetic Maintenance Data: A Retrospective Analysis Study. *JPO Journal of Prosthetics and Orthotics*10.1097/JPO.0000000000000400, (2022).

[34] Exceed Research Network and ISPO. Ethical Considerations and Approaches for Conducting Clinical Research Studies related to Prosthetics, Orthotics and Wheelchair Technology in the Low- and Middle-Income Countries. <https://eprints.soton.ac.uk/449284/1/ethical_considerations_to_po.pdf. (2021).

[35] Bhatnagar, T., Bandukda, M., Bell, D. & Holloway, C. in *The 4th African Human Computer Interaction Conference* 190–200 (Association for Computing Machinery, East London, South Africa, 2023).

[36] Williams, R. J., Takashima, A., Ogata, T. & Holloway, C. A pilot study towards long-term thermal comfort research for lower-limb prosthesis wearers. *Prosthet. Orthot. Int.* **43**, 47–54. 10.1177/0309364618791604 (2019). 30080114 10.1177/0309364618791604

[37] Saradjian, A., Thompson, A. & Datta, D. The experience of men using an upper limb prosthesis following amputation: Positive coping and minimizing feeling different. *Disabil. Rehabil.* **30**, 871–883. 10.1080/09638280701427386 (2008). 17852212 10.1080/09638280701427386

