# Supplementary material for: Introducing an adjustable upper limb prosthesis into a Ugandan clinical service: impacts on free living behaviour and prosthetic use
Source: Sci Rep. 2025 Apr 4;15:11585. doi: 10.1038/s41598-025-96779-w (PMC11971239; doi:10.1038/s41598-025-96779-w)
Supplement: Supplementary file 1 — Supplementary Information. [file 41598_2025_96779_MOESM1_ESM.docx]

# Appendix one: Semi-structured interview

**Introduction**

- - Thank the participant.
  - Briefly introduce yourself.
  - Briefly explain the aims of the study: show information sheet, explain how the information is treated (confidentiality and anonymity) discuss concerns, questions, etc.
  - Explain that there is no right or wrong answers, and we are interested in their view and experiences
  - Ask for explicit permission to record the conversation.
  - Obtain informed consent.
  - Ask about any questions again and whether they have doubts or concerns once again

**Interview**

1. Could you tell us about your experience (if any) of using an upper limb prosthesis before this study

*Most prosthetic sockets look like this (show photo or example of traditional one-piece) socket. We prescribed you a socket (show photo, or an example) which allowed you to tighten or loosen it, as needed.*

1. Did you continue to wear your prosthesis?
   1. If so, please tell us why?
   2. Do you wear your device when around the house?
      1. If not, please tell us why?
2. Can you tell us what you thought of the socket?
   1. What factors influenced when you wore it (if at all) or when you choose not to wear it? [*Please illustrate with some examples*]
   2. Did you clean your socket?
3. Could you tell us what you thought of the prosthetic hands you were given?
   1. What examples of ADLs )if any) did you with the hands
   2. What challenges did you generally find with the hands you were fitted with?
   3. What did you think about the wrist joints?
4. How did wearing the prosthesis affect your social interaction/community integration?
   1. How about your family members, what do they think about your hand?
5. Did you use the Limb Buddy service?
   1. If ***yes***, tell us about your experiences, both positive and negative
6. If the socket needed repairing (either in the past) or in the future, would you/did you do, and why?
7. Please describe an ideal repair service. [*Where would it be? Who would run it? How would you like to contact it?*]
8. How did you find wearing the monitors?
   1. Were there occasions when you felt like taking one or more of the monitors off?
   2. If so, why was that?
9. How did you did you find filling the dairy?
10. The study has ended. What kind of support do you expect from the people who fitted you with the hand?
11. Say the Koalaa system was for sale, would you buy it? How much?
12. Would you like to add any further information to what we have discussed?
13. Thank you for your time

# Appendix two: Synchronisation Test Between Axivity and activPAL monitors

To ensure that the data obtained from the two different activity monitors (Axivity and activPAL) could be combined and compared at the same time point (for up to a week), a synchronisation test was performed to evaluate the relative drift (if any) between the two monitors. This test involved an Axivity monitor being taped to an activPAL monitor, and the pair of monitors being moved from a horizontal to vertical orientation (and back again) multiple times per day across the length of the recording. This test was conducted for four days with four different pairs of monitors.

To extract the time of each change of orientation for the Axivity monitor, the data visualisation in OMGUI was used, which showed the acceleration value for each of the principal axes of the monitor. The point of orientation change could be easily identified, with a call out note enabling the timestamp of that point to be easily read (when zoomed in) (see Supplementary Figure 1 below).


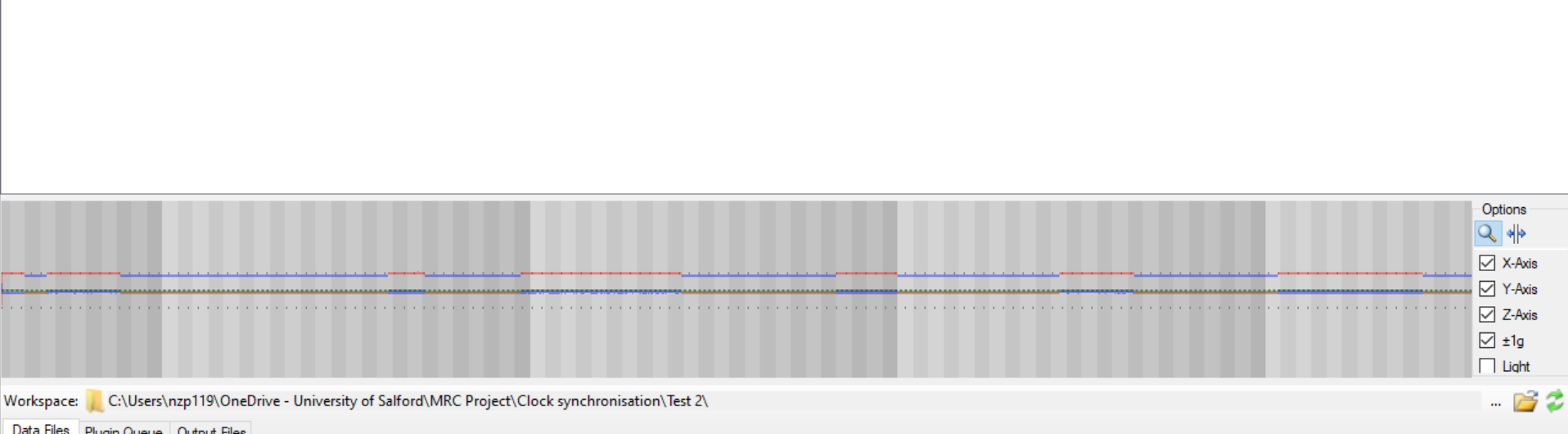

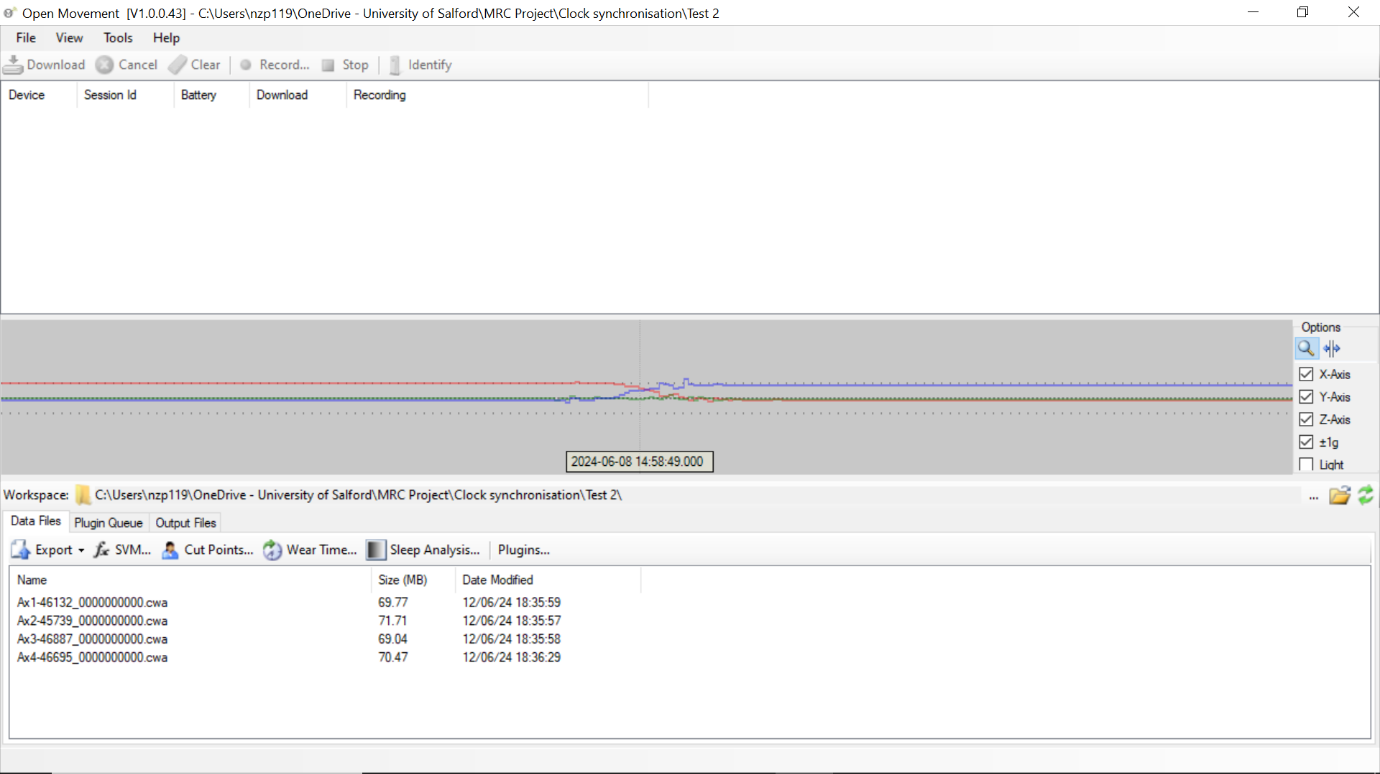


Supplementary Figure 1: Axivity monitor data viewed in OMGUI, with zoomed in section at point of orientation change.

To extract the equivalent time point from the activPAL data, the compressed accelerometer export was used, which shows the accelerometer readings for the activPAL sensor, but only at points at which significant change in acceleration occurs. The time points were identified by finding the time stamp of values which had 10+ readings in the export, which excludes incidental acceleration values which may have been detected as a result of knocking the table the monitors were resting on etc (see Supplementary Figure 2 below).


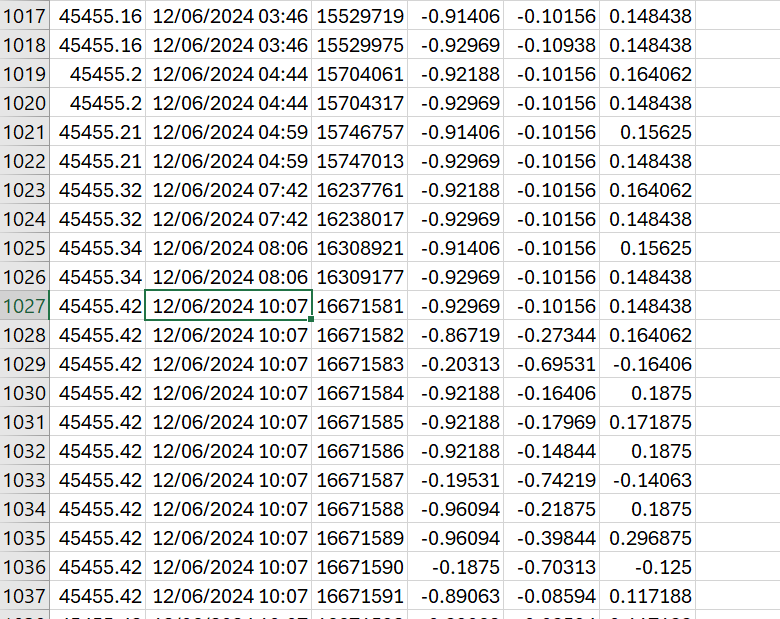


Supplementary Figure 2: Acceleration export from PALAnalysis, column 1 and 2 represent the time stamp, and columns 3-5 show the acceleration values. The highlighted cell shows an extracted data point where the orientation was deemed to have changed. The values above were excluded due to a small number of data points at their time stamps (1/2/3)

These two sets of data, from each pair of Axivity and activPAL monitors, were plotted against each other, with the activPAL timestamp on the x-axis and the Axivity timestamp on the y-axis (see Supplementary Figure 3 below). An x=y line was also added to the plot to show where these points should lie if synchronisation was perfect.

Supplementary Figure 3: Axivity vs activPAL timestamps plotted against each other to demonstrate synchronisation.

The timestamps extracted from each of these monitors can also be viewed in table format (Supplementary Table 1 below). From this it can be seen that after approximately 4 days of data collection the drift between the two monitors was a maximum of 7 minutes, which is deemed acceptable.

Supplementary Table 1: Full dataset of equivalent timestamps for activPAL and Axivity monitors. Monitors of the same number (i.e. 1, 2 etc. were taped together).

| ActivPAL 1 | 08/06/24 14:58 | 08/06/24 16:26 | 08/06/24 21:15 | 09/06/24 14:43 | 09/06/24 17:05 | 09/06/24 23:16 | 10/06/24 09:46 | 10/06/24 19:54 | 10/06/24 23:53 | 11/06/24 10:26 | 11/06/24 15:18 | 12/06/24 00:40 | 12/06/24 10:06 |
| --- | --- | --- | --- | --- | --- | --- | --- | --- | --- | --- | --- | --- | --- |
| ActivPAL 2 | 08/06/24 14:58 | 08/06/24 16:26 | 08/06/24 21:15 | 09/06/24 14:43 | 09/06/24 17:06 | 09/06/24 23:18 | 10/06/24 09:47 | 10/06/24 19:56 | 10/06/24 23:54 | 11/06/24 10:26 | 11/06/24 15:19 | 12/06/24 00:41 | 12/06/24 10:07 |
| ActivPAL 3 | 08/06/24 14:58 | 08/06/24 16:25 | 08/06/24 21:14 | 09/06/24 14:42 | 09/06/24 17:05 | 09/06/24 23:17 | 10/06/24 09:45 | 10/06/24 19:54 | 10/06/24 23:53 | 11/06/24 10:27 | 11/06/24 15:19 | 12/06/24 00:41 | 12/06/24 10:06 |
| ActivPAL 4 | 08/06/24 14:58 | 08/06/24 16:26 | 08/06/24 21:14 | 09/06/24 14:43 | 09/06/24 17:06 | 09/06/24 23:18 | 10/06/24 09:47 | 10/06/24 19:55 | 10/06/24 23:55 | 11/06/24 10:28 | 11/06/24 15:20 | 12/06/24 00:41 | 12/06/24 10:07 |
|  |  |  |  |  |  |  |  |  |  |  |  |  |  |
| Axivity 1 | 08/06/24 14:58 | 08/06/24 16:26 | 08/06/24 21:16 | 09/06/24 14:45 | 09/06/24 17:08 | 09/06/24 23:20 | 10/06/24 09:50 | 10/06/24 19:59 | 10/06/24 23:58 | 11/06/24 10:32 | 11/06/24 15:25 | 12/06/24 00:47 | 12/06/24 10:13 |
| Axivity 2 | 08/06/24 14:58 | 08/06/24 16:26 | 08/06/24 21:16 | 09/06/24 14:45 | 09/06/24 17:08 | 09/06/24 23:20 | 10/06/24 09:50 | 10/06/24 19:59 | 10/06/24 23:59 | 11/06/24 10:32 | 11/06/24 15:25 | 12/06/24 00:47 | 12/06/24 10:13 |
| Axivity 3 | 08/06/24 14:58 | 08/06/24 16:26 | 08/06/24 21:16 | 09/06/24 14:45 | 09/06/24 17:08 | 09/06/24 23:20 | 10/06/24 09:50 | 10/06/24 19:59 | 10/06/24 23:58 | 11/06/24 10:32 | 11/06/24 15:25 | 12/06/24 00:47 | 12/06/24 10:13 |
| Axivity 4 | 08/06/24 14:58 | 08/06/24 16:26 | 08/06/24 21:16 | 09/06/24 14:45 | 09/06/24 17:08 | 09/06/24 23:20 | 10/06/24 09:50 | 10/06/24 19:59 | 10/06/24 23:58 | 11/06/24 10:32 | 11/06/24 15:25 | 12/06/24 00:47 | 12/06/24 10:13 |

Appendix three: Physical Activity and prosthesis wear data for each participant

Supplementary Figures 5 and 6 show the activPAL data at T0, T1, and T2 projected onto spirals, overlaid (at the edges) with prosthesis wear periods. The benefit of these spiral plots over linear visualisation is that activity can be continuously observed across the midnight point. Each loop of the spiral represents 24 hours, with midnight at the top and midday at the bottom and data capture beginning in the centre. Colours within the spirals represent the activity classification (e.g. sitting, standing, walking etc.). Periods of prosthesis wear are indicated by black banding along the edge of the spiral. Where activPAL data were unavailable, prosthesis wear periods are still shown without the overlaid activPAL data. A star (*) symbol to the top left of the spiral indicates that the prosthesis worn in the recording period was the one they entered the study with. In cases where prosthesis wear is reported but without the star symbol, this indicates that the prosthesis worn was the Koalaa. The figure in red (top right of each spiral) indicates the average number of steps/day over the recording period. The blue figure (bottom right of each spiral) indicates the average prosthesis wear time (hours/day) over the recording period.

To aid the reader’s understanding of these plots, an example of one such spiral is discussed below (Supplementary Figure 4: P8, T1). As the participant entered the study without a prosthesis, no star symbol is shown. The recording started around 12pm on a Tuesday (innermost spiral) when the participant was wearing their Koalaa prosthesis. Moving clockwise around the spiral we can see the participant wore their prosthesis while a. alternating between short periods of stepping and extended periods of sedentary behaviours until about 6:30pm. They then doffed their prosthesis and showed sedentary behaviour until 11:30pm, when they went to sleep (primary lying). The following day they woke up around 8am, donning their prosthesis shortly after and showed stepping behaviour until just before 9am. The period up to midday was predominantly sedentary (end of first 24-hour period). Subsequent days of the recording period showed similarly high levels of prosthesis wear, typically donning the prosthesis shortly after waking (~9am) and removing it in the mid-late evening. Physical behaviours during the day were typically a mix of sedentary and stepping, with occasional periods of seated transport. Over the recording period we observed an average of 9937 steps/day and the prosthesis was worn for an average of 11.67 hours/day.


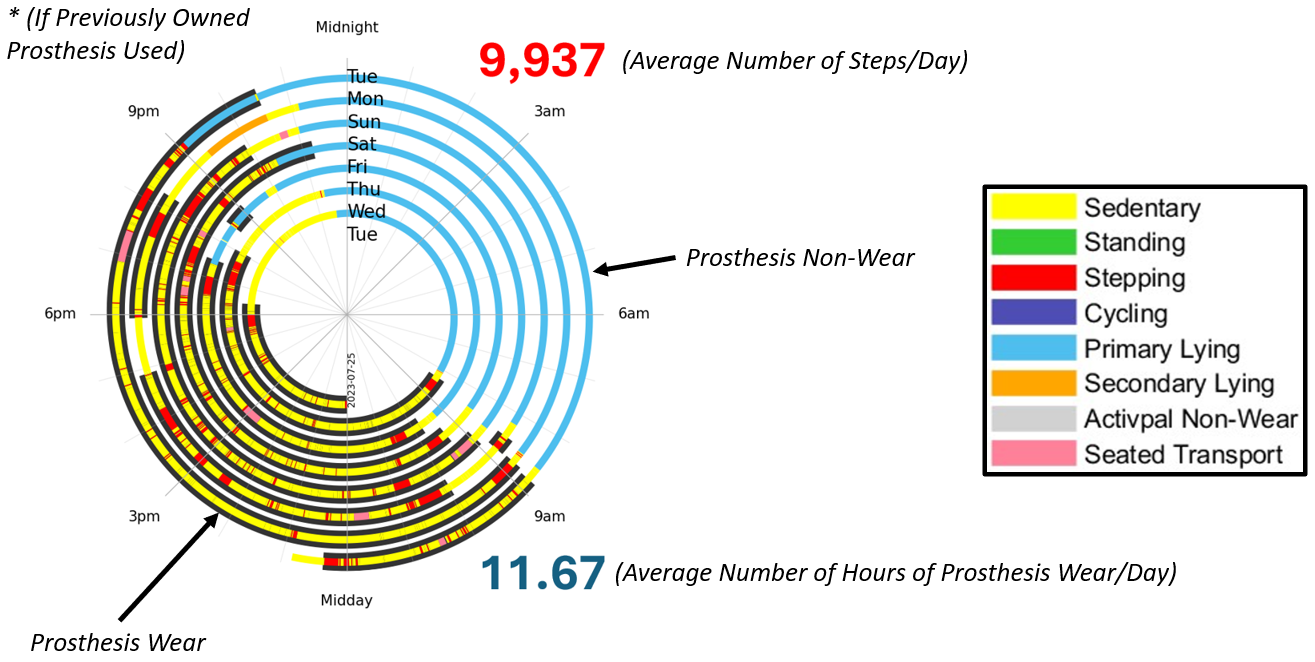

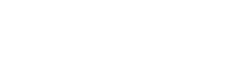


Supplementary Figure 4: Example data (P8 T1) to assist readers’ interpretation of Supplementary Figures 5 and 6. Each loop of the spiral represents 24 hours, with midnight at the top and midday at the bottom. Colours within the spirals represent the activity classification (see colour key), and outer bands (dark border or white border) represent prosthesis wear and non-wear respectively.

Supplementary Figure 5: Activity and prosthesis wear data for the four participants with prior experience of wearing a prosthesis (P1,P2,P3,P8)

**9.15**


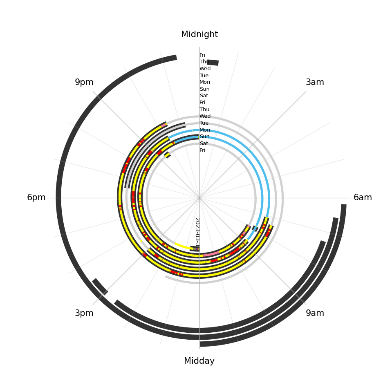

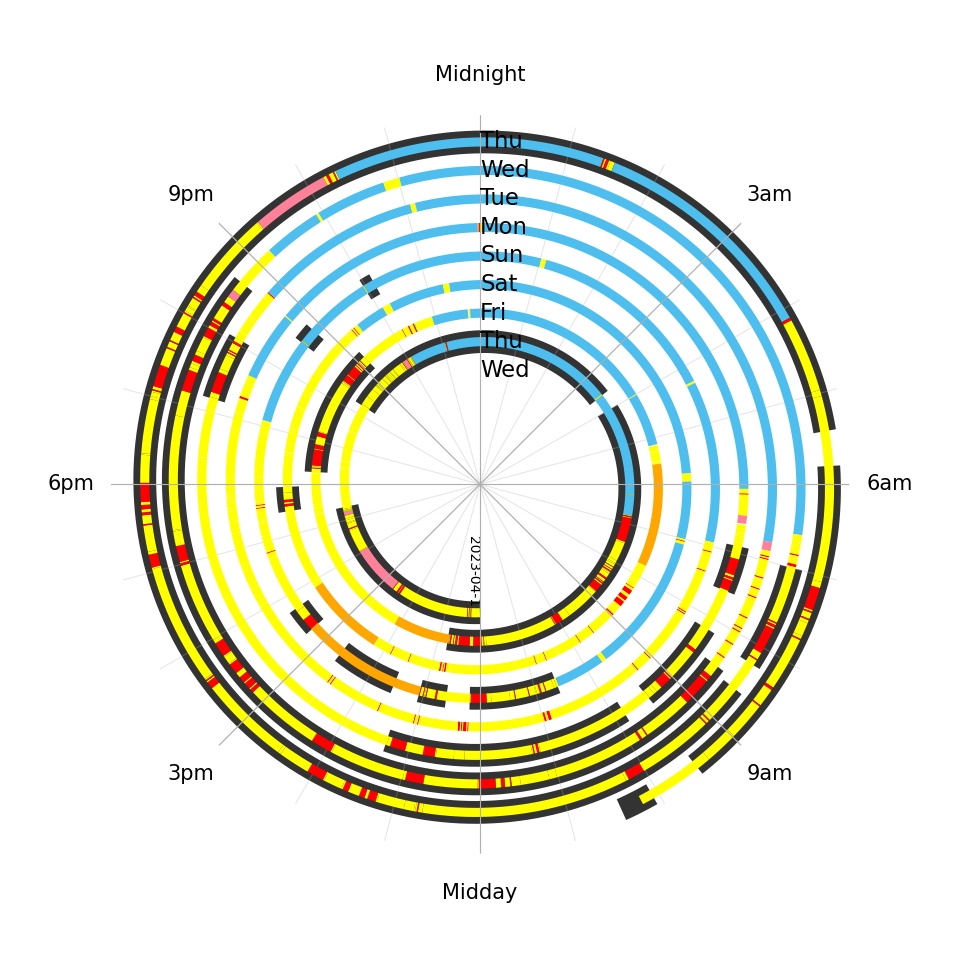

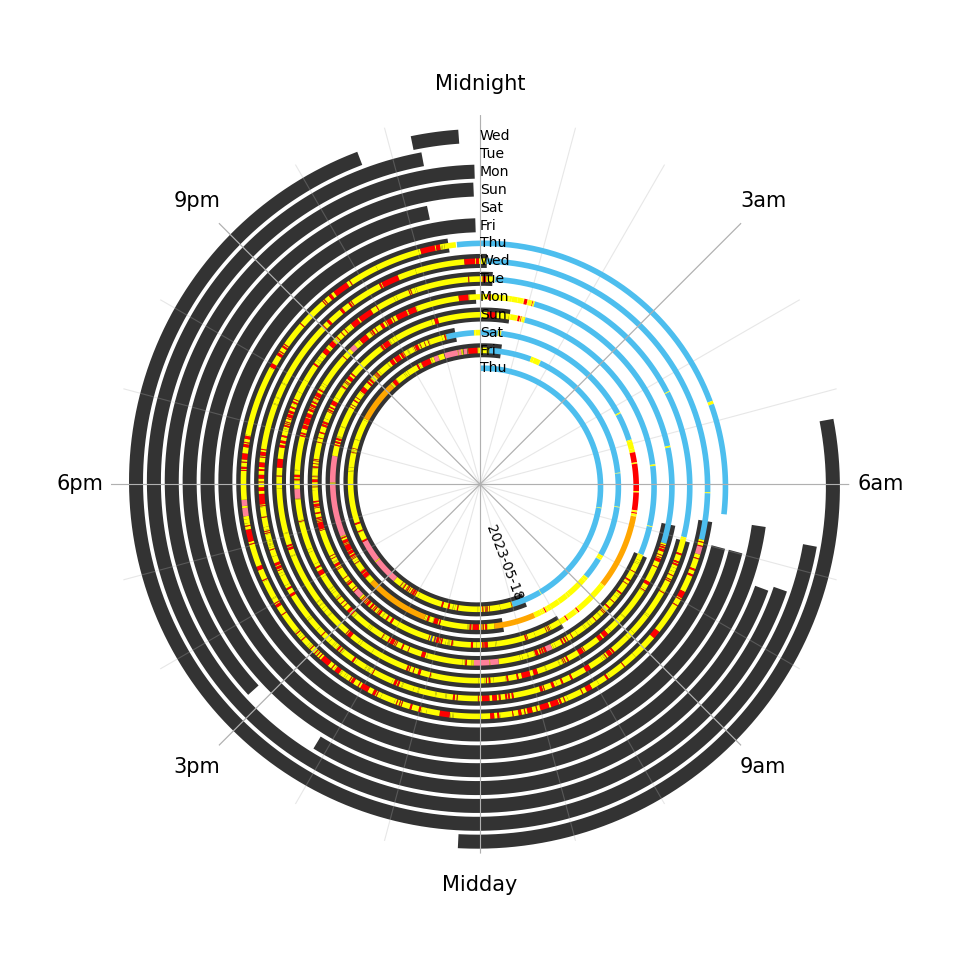

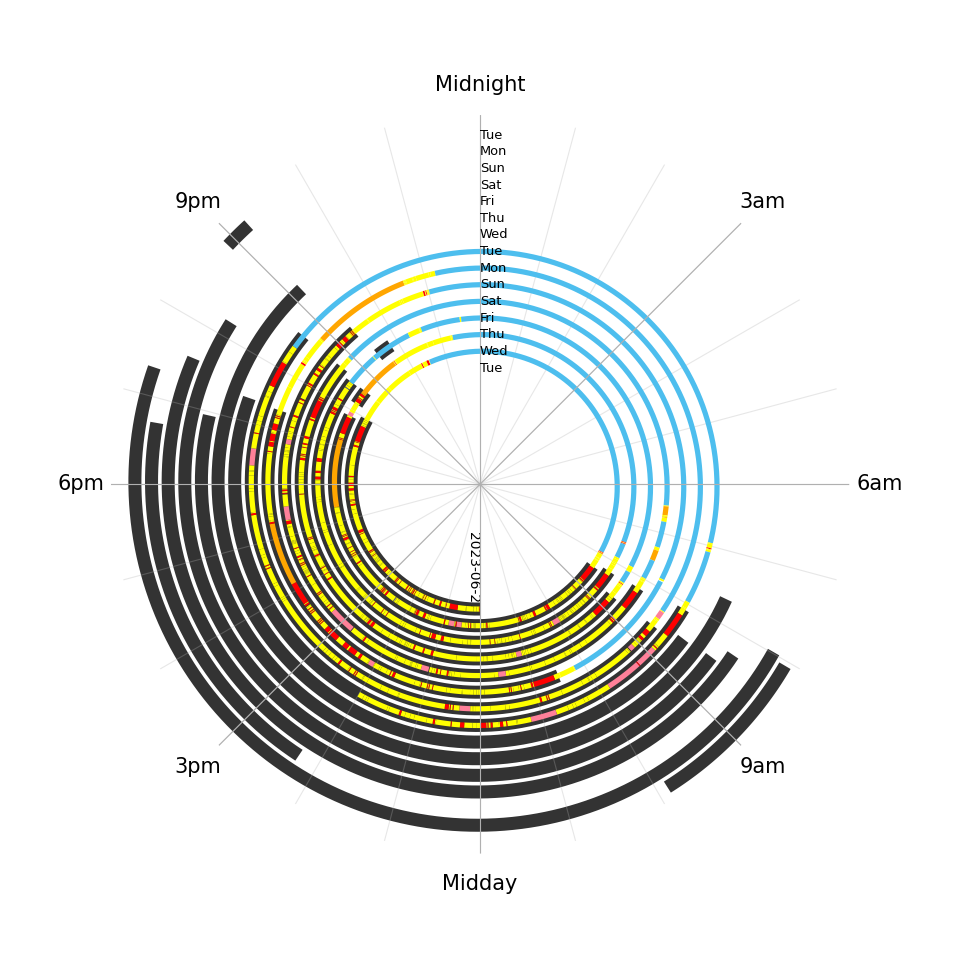

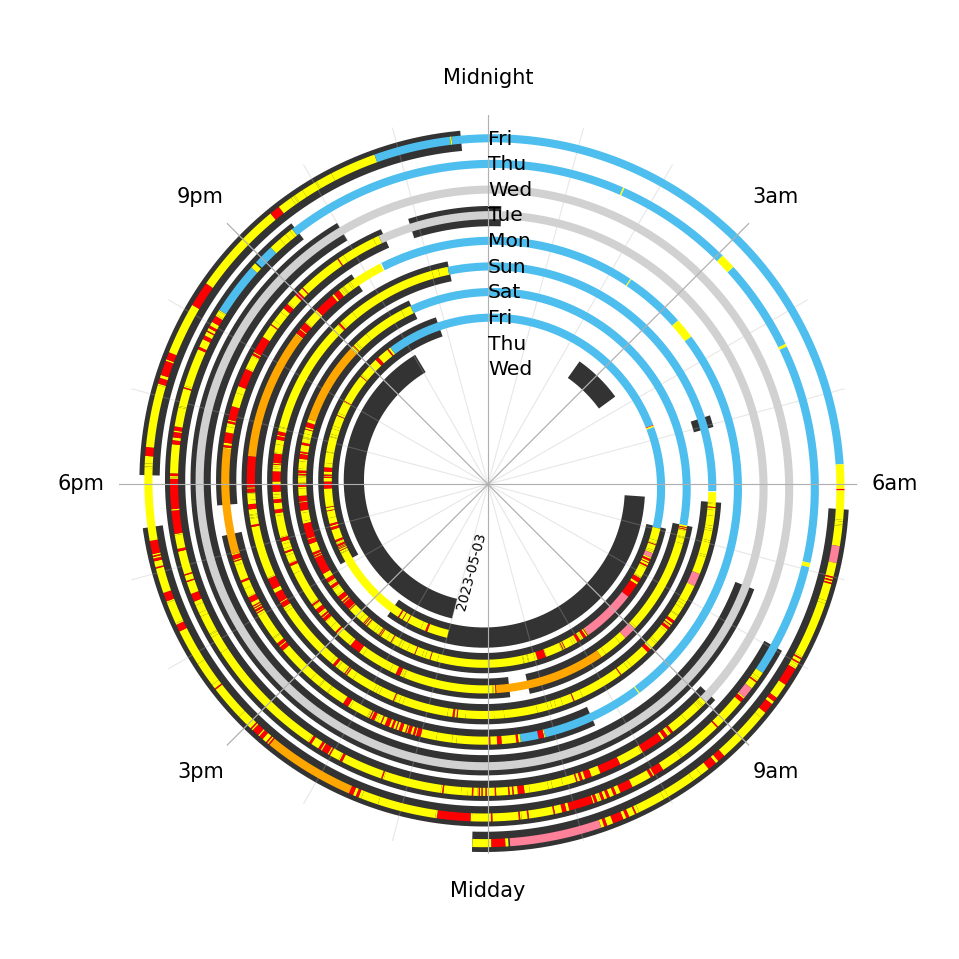

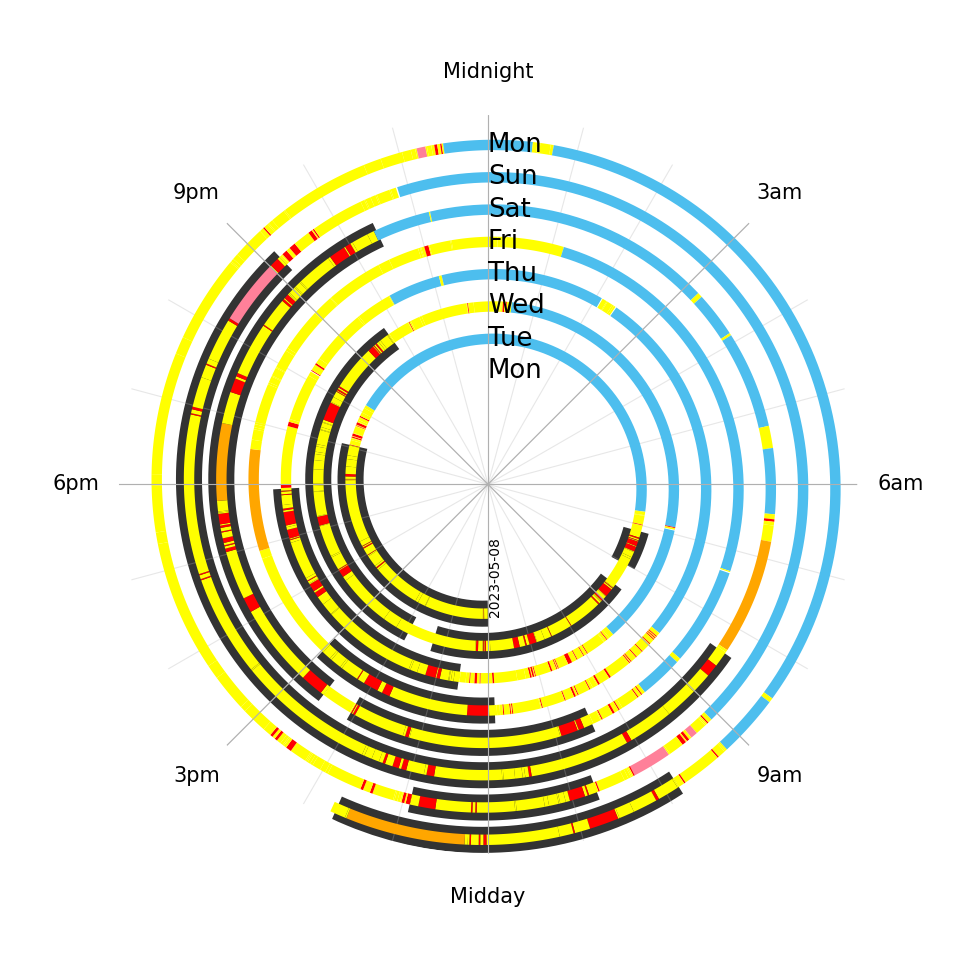

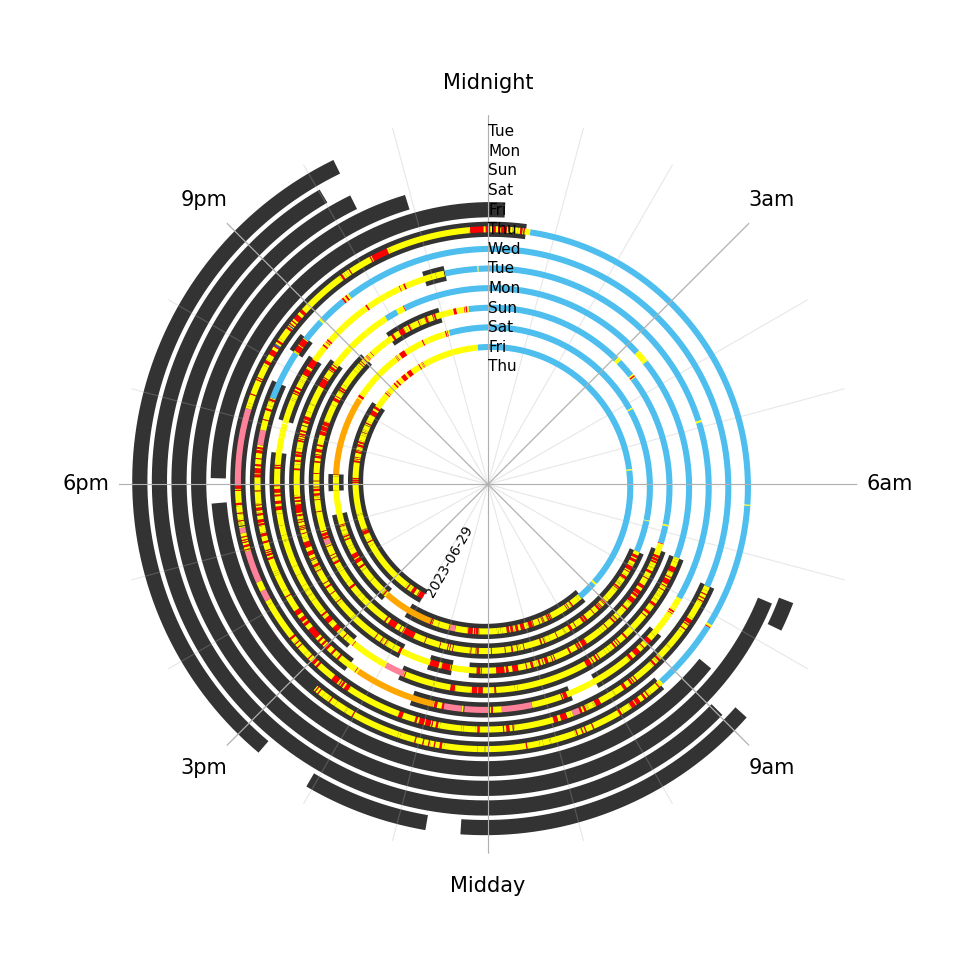

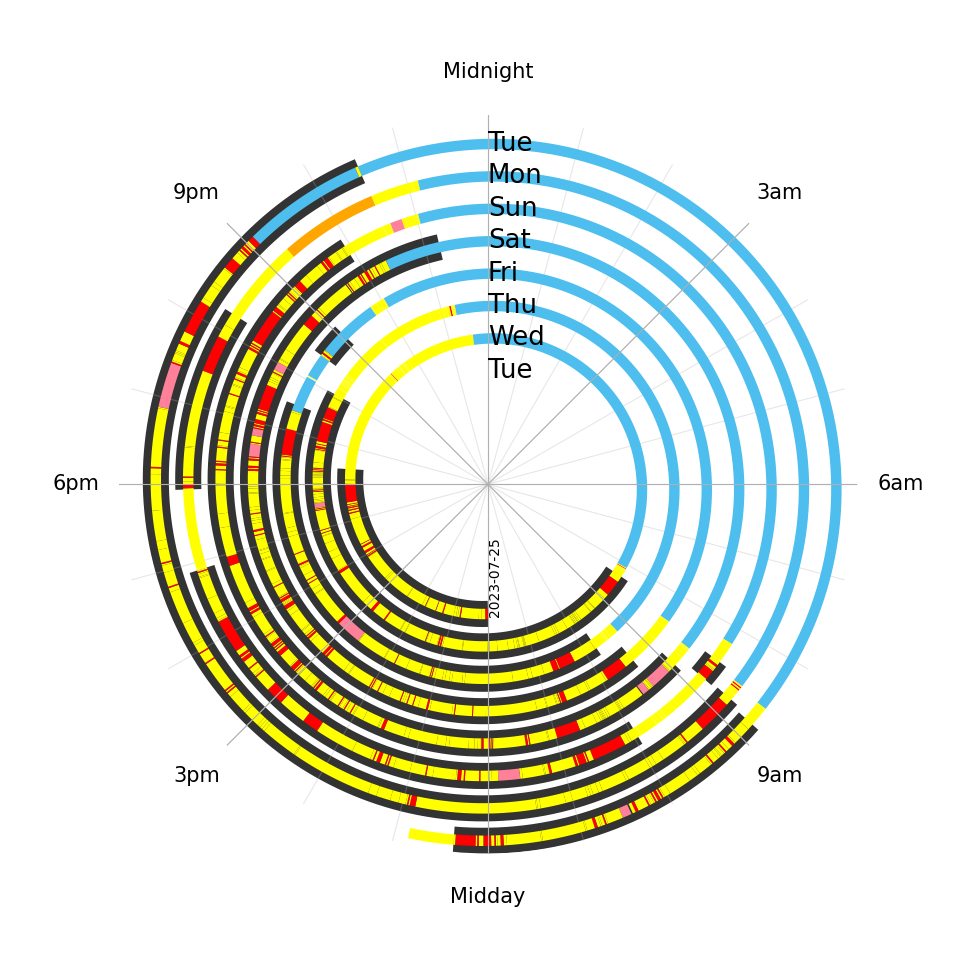

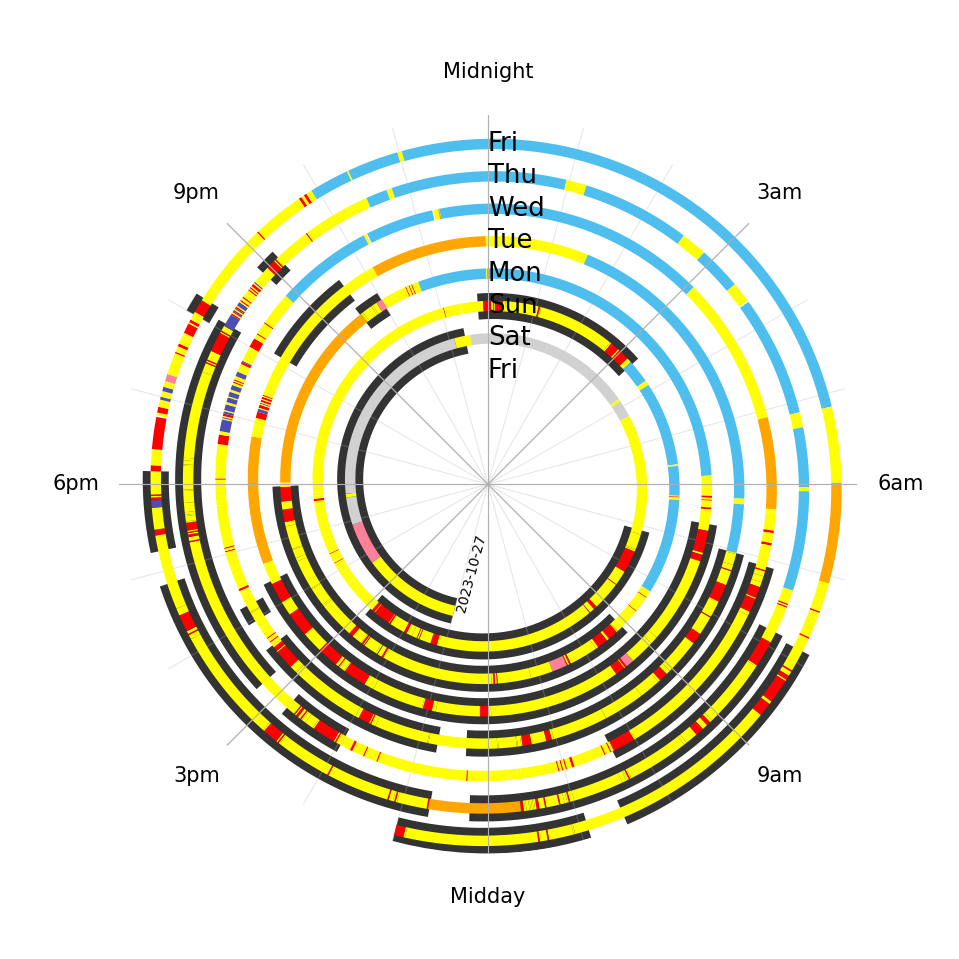

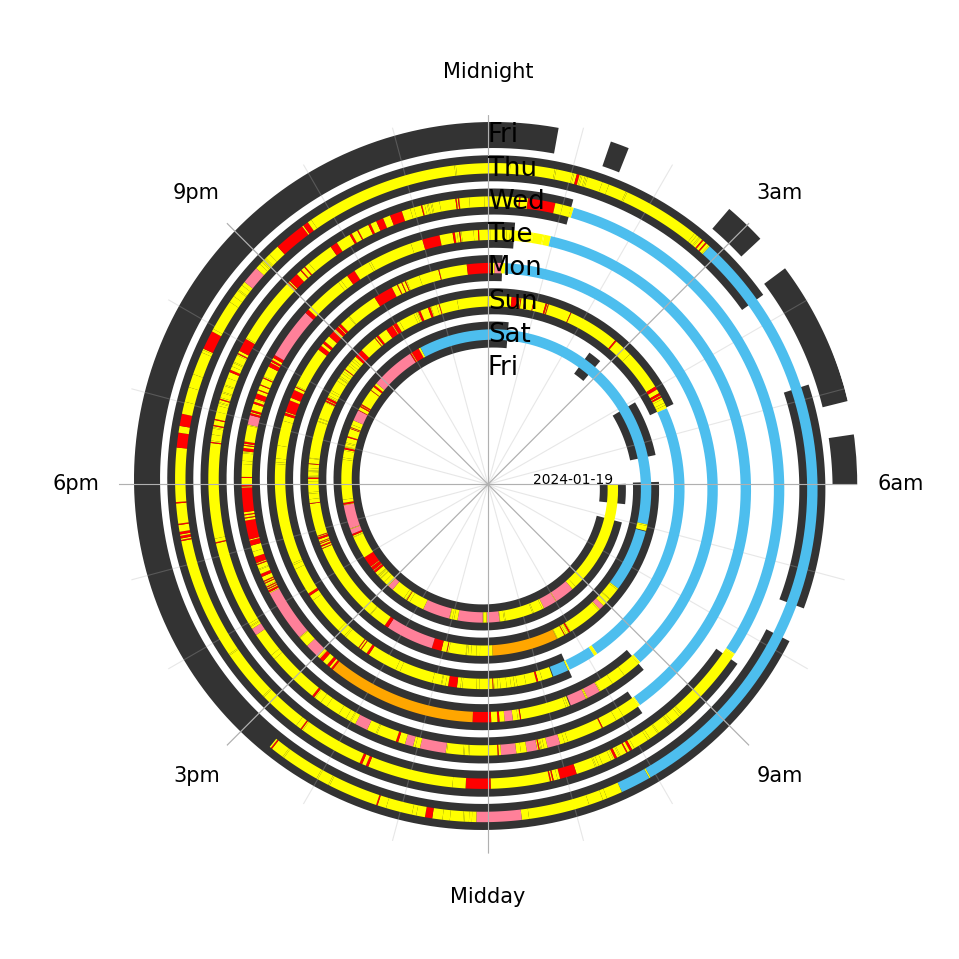

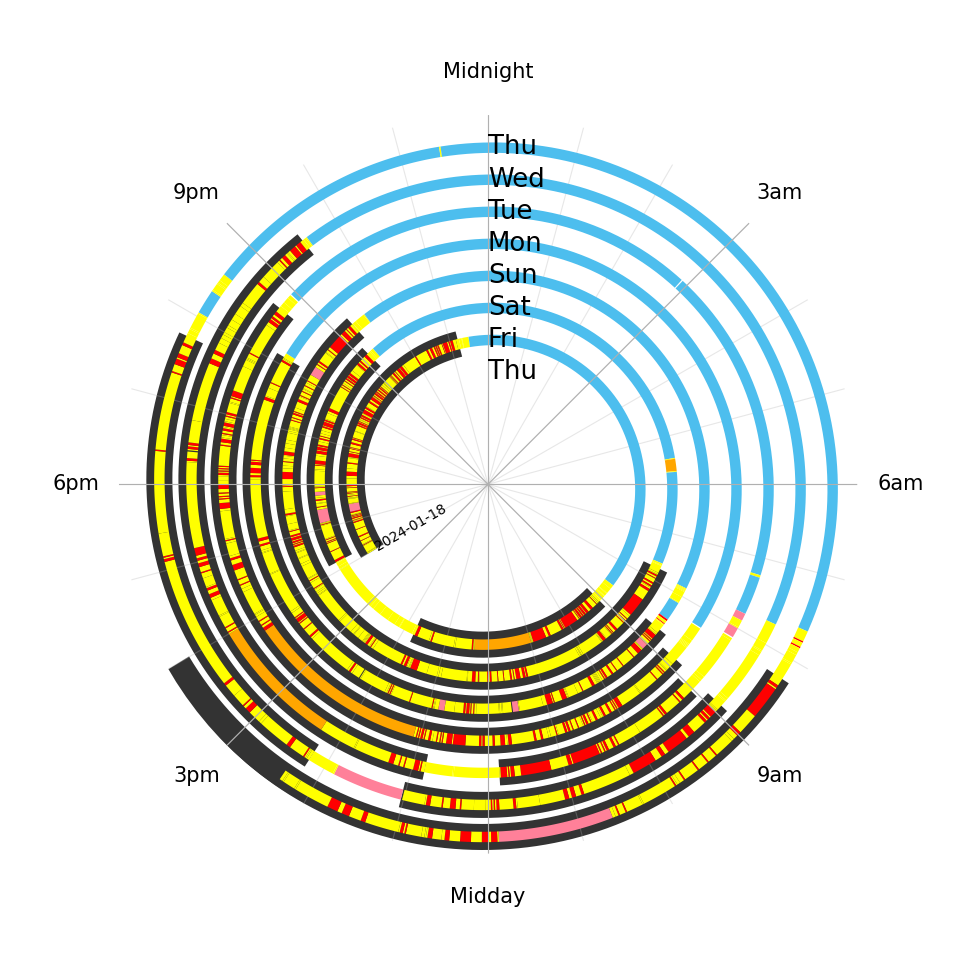


T0

T1

T2

Participant 1

Participant 2

Participant 3

Participant 8

**10,007**

**6.38**

**9,105**

**11.06**

**15,022**

**15.84**

**7,830**

**9.15**

**11,755**

**14.93**

**9,937**

**11.67**

**14,285**

**12.75**

**7,048**

**8.40**

**11,810**

**11.81**

**10,038**

**18.27**

**9,524**

**10.61**

*

*

*

*

*

*

Supplementary Figure 6: Activity and prosthesis wear data for the four participants with no prior experience of wearing a prosthesis (P4,P5,P6,P7)


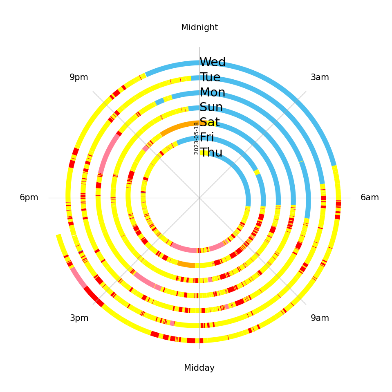

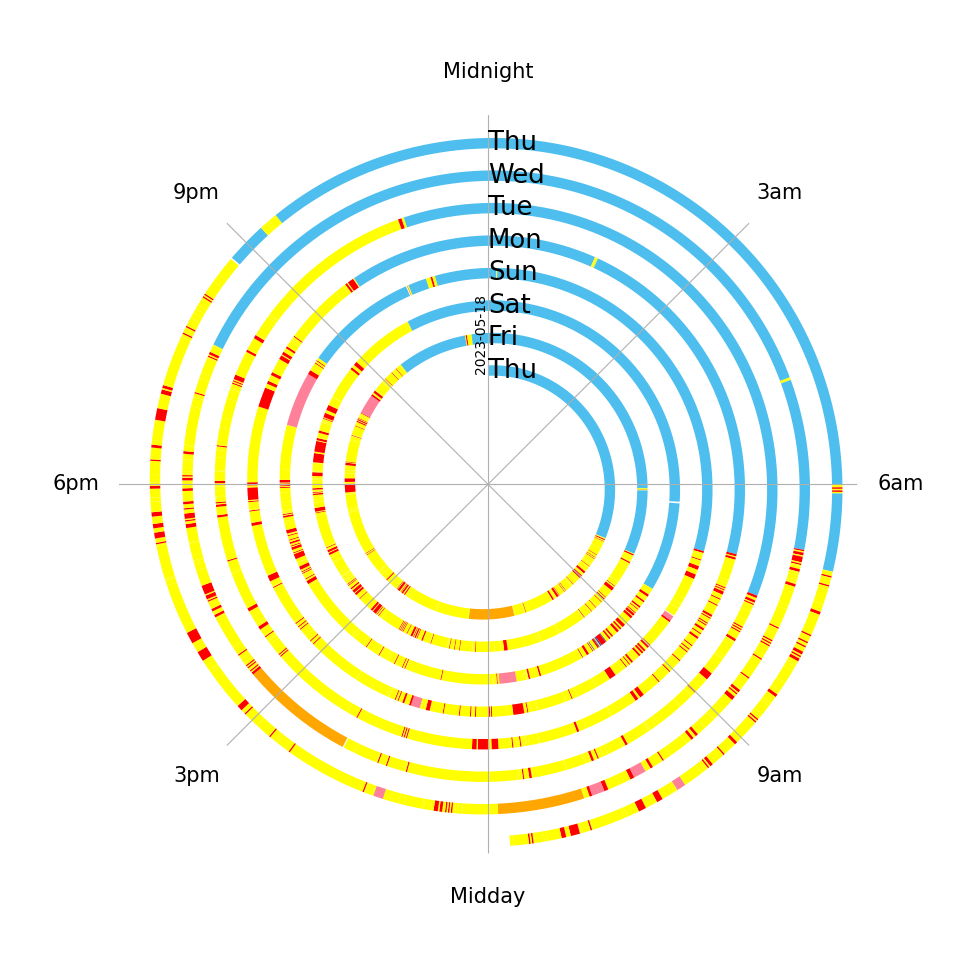

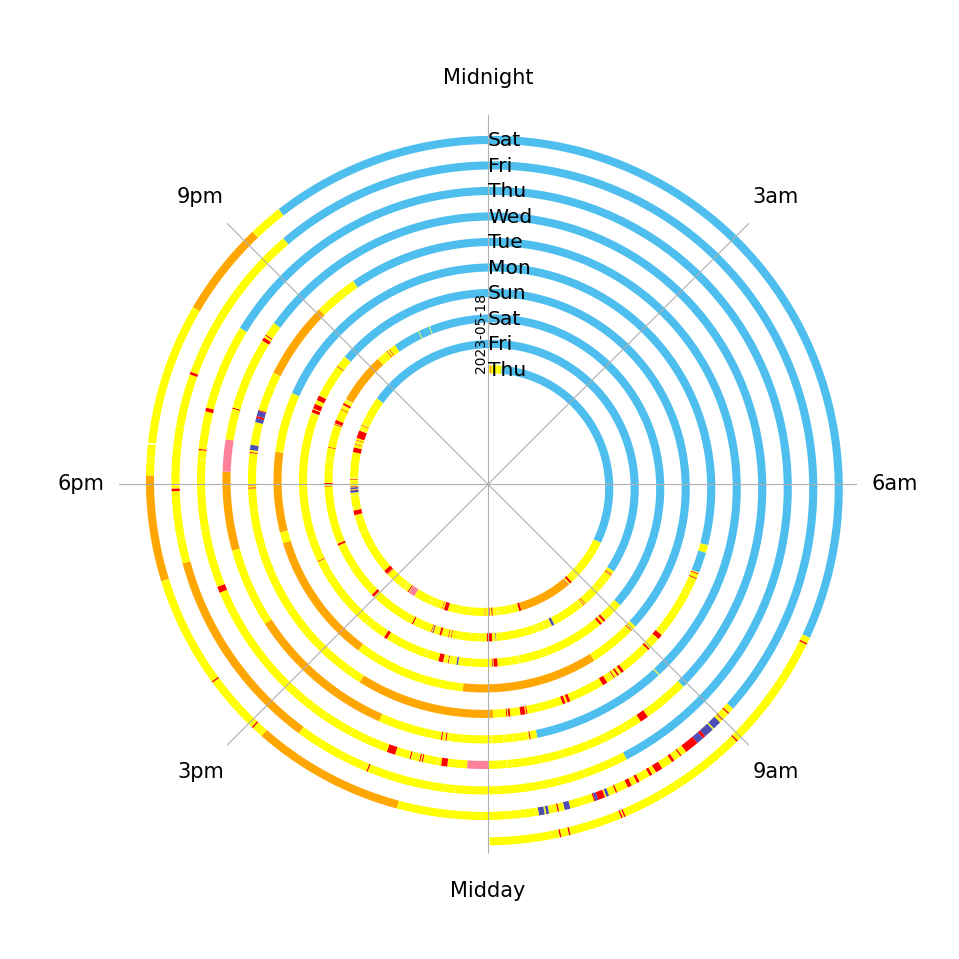

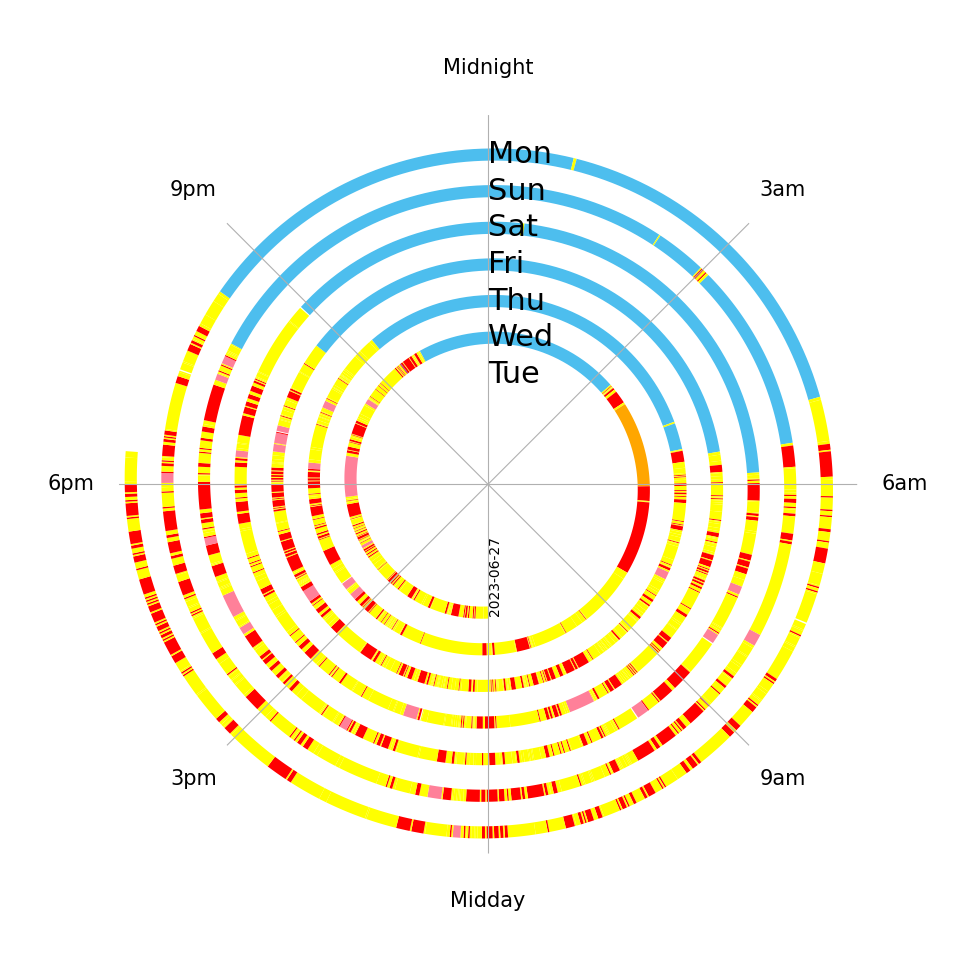

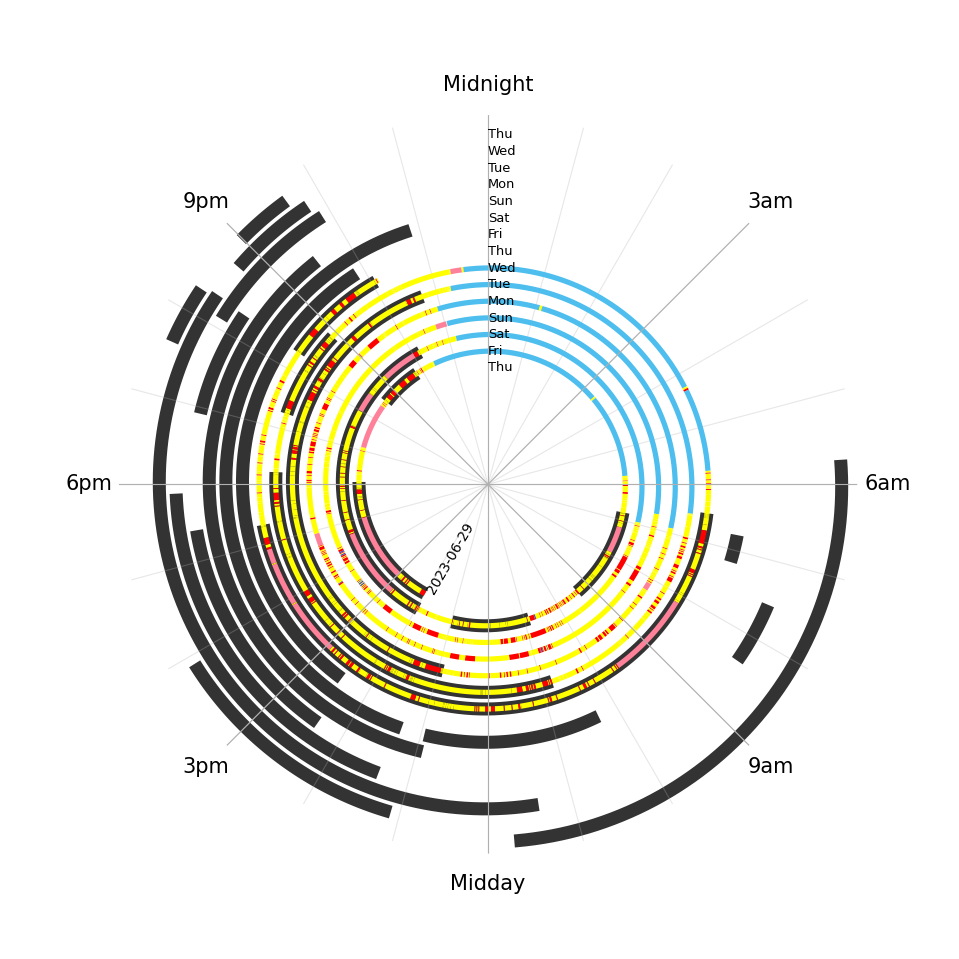

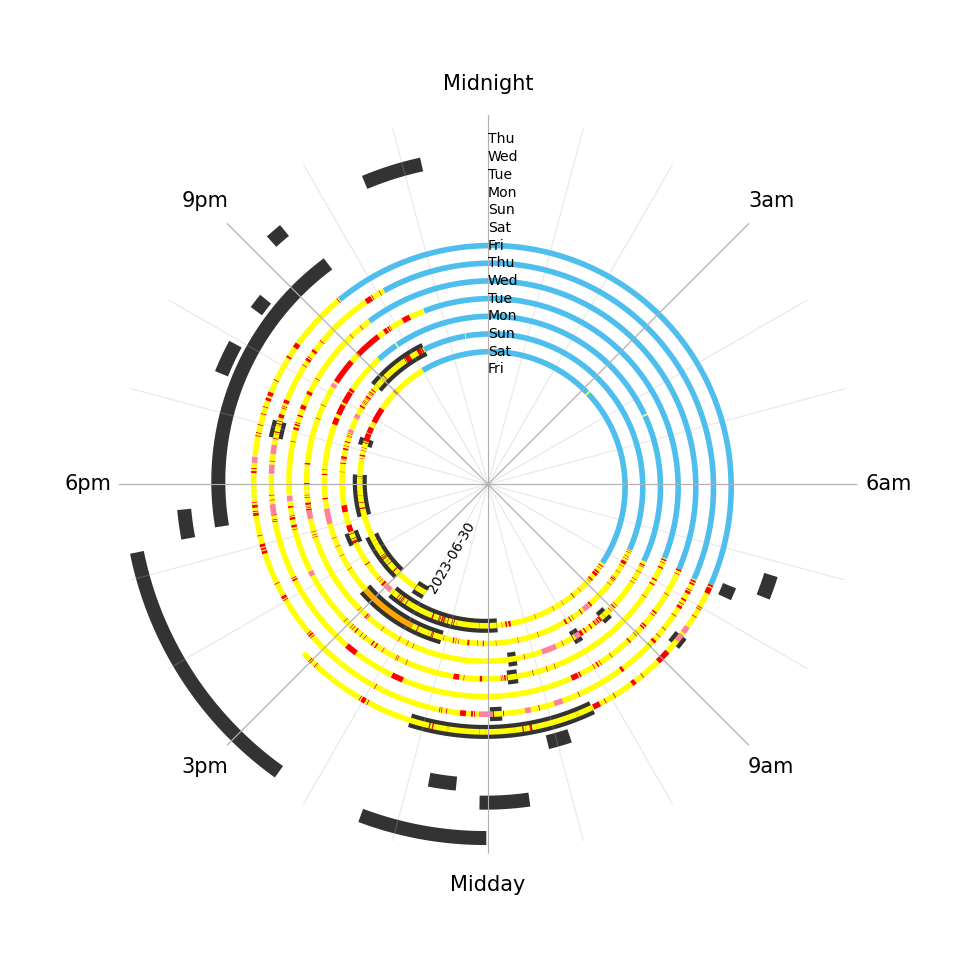

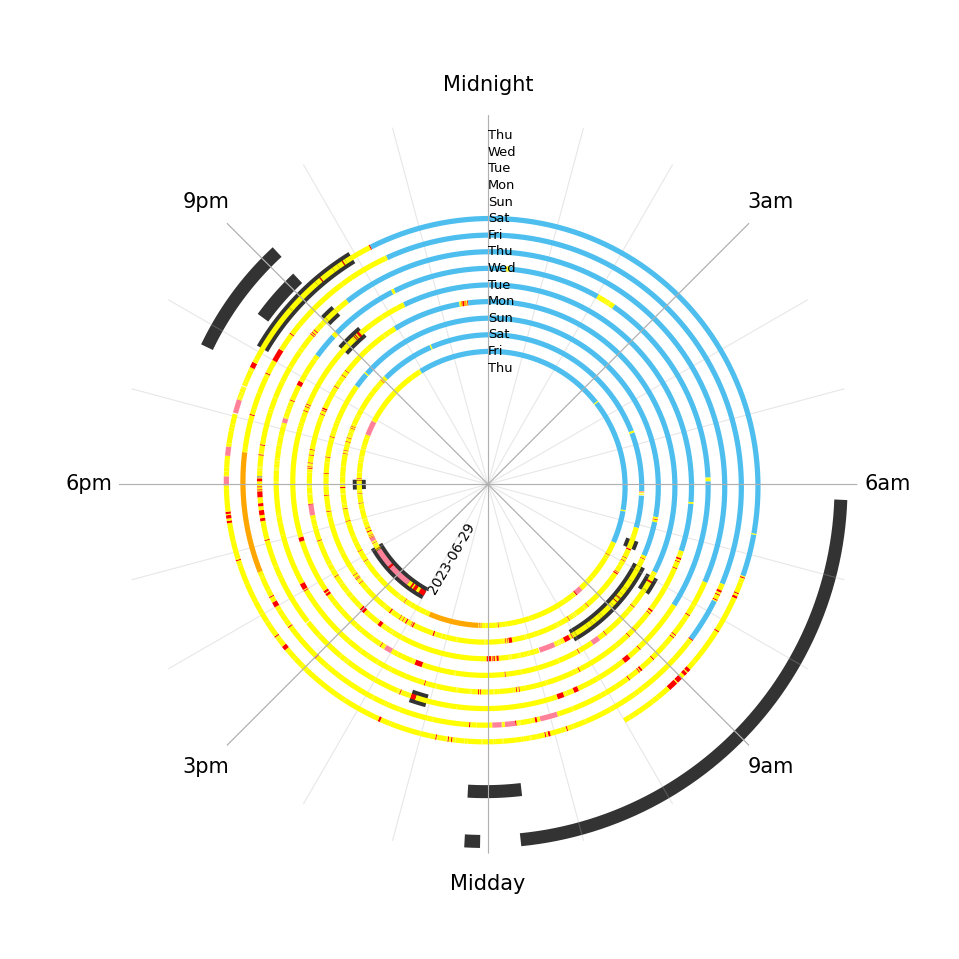

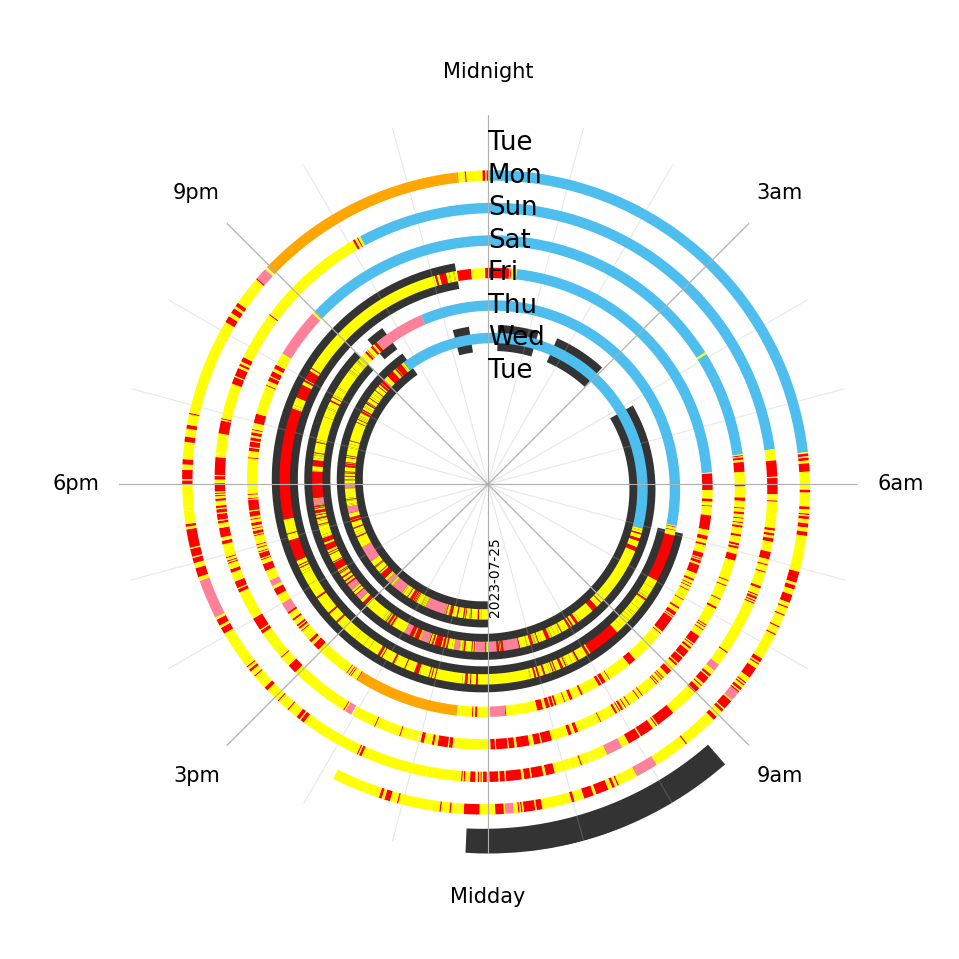

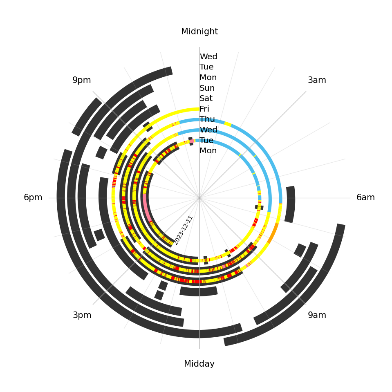

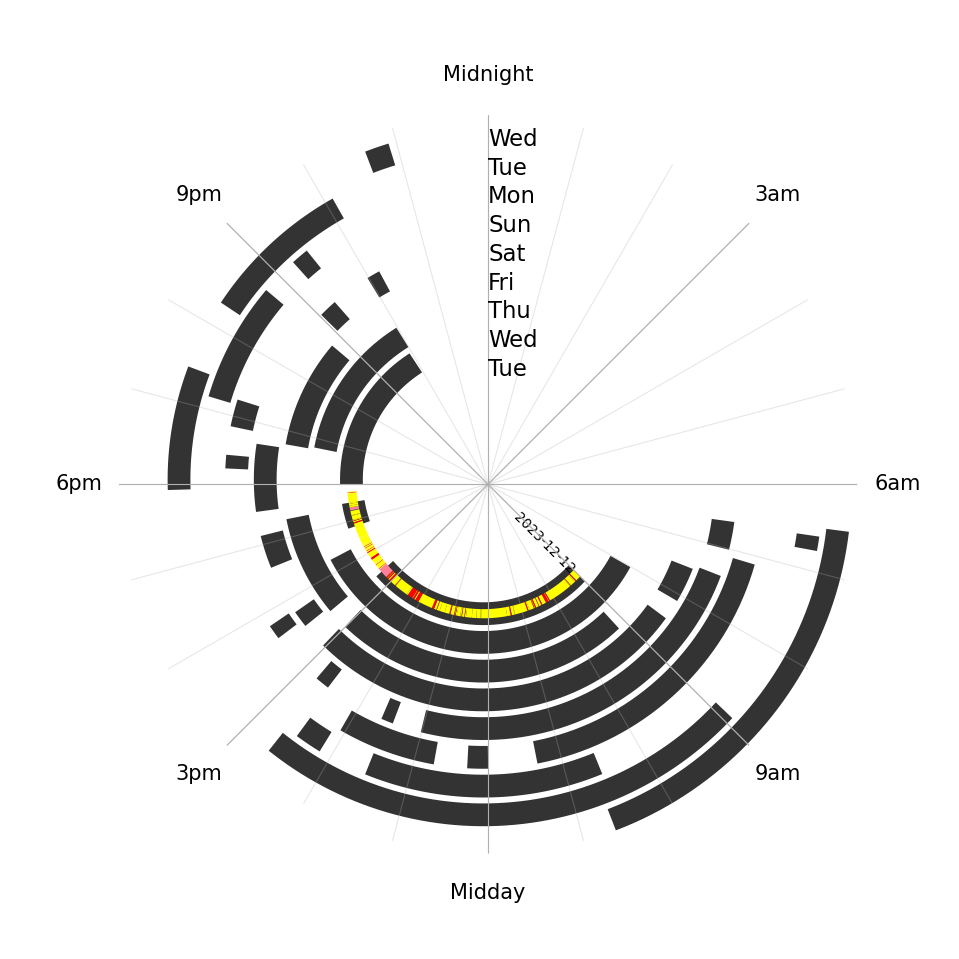

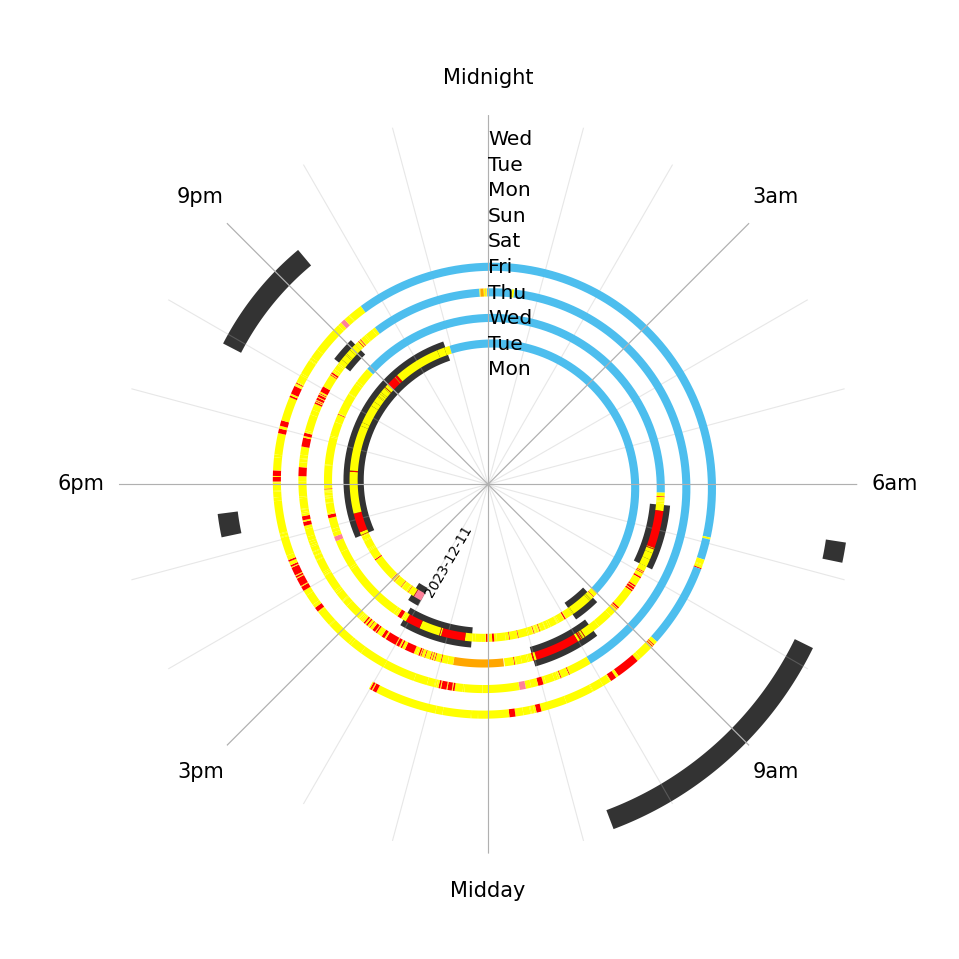

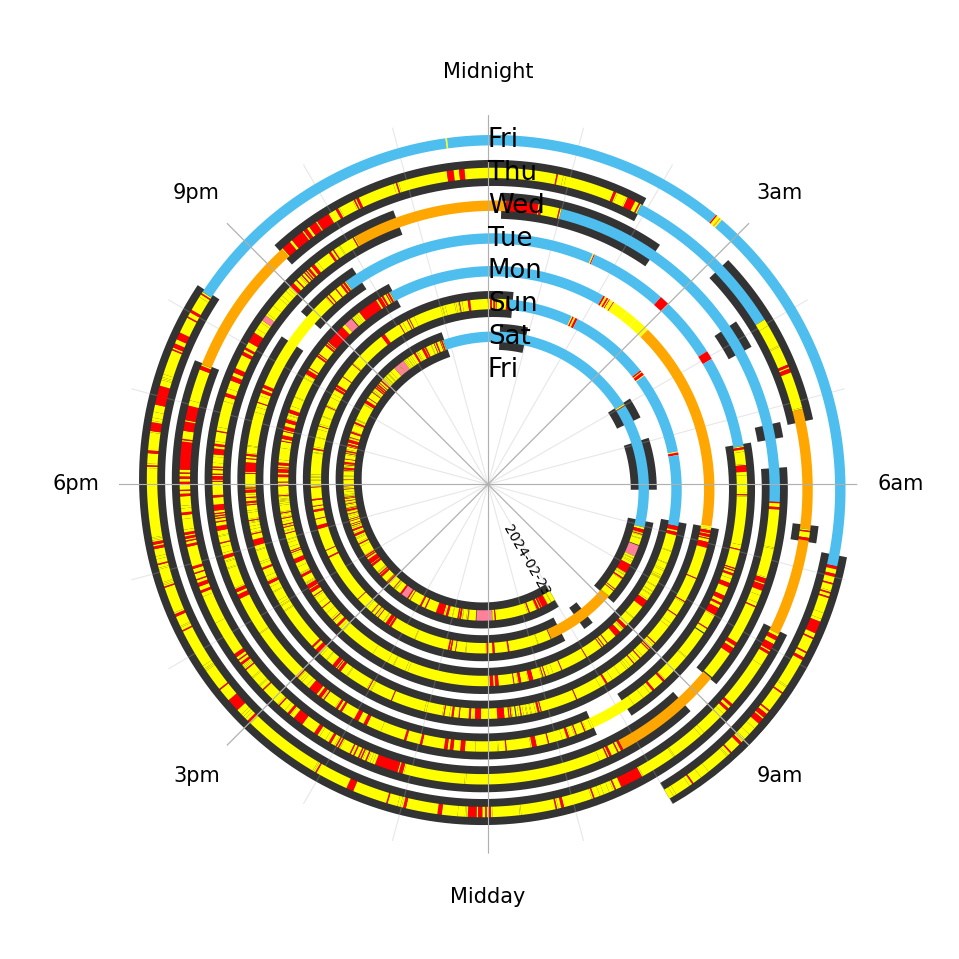


Participant 4

Participant 5

Participant 6

Participant 7

T0

T1

T2

**16,389**

**21,128**

**3,060**

**9,048**

**14,631**

**7.90**

**22,491**

**7.01**

**4,408**

**1.18**

**9,621**

**2.04**

**14,926**

**9.72**

**13,845**

**16.80**

**11,935**

**1.94**

**17,881**

**9.14**

As can be seen above, the duration of the ActivPal recording period varied considerably between participants. As mentioned in the paper, this was due to battery charging issues and monitor failure. The Axivity recording period for each participant was never less than 7 days. Below (Supplementary Table 2), for each participant and each of the three data collection periods, we detail the durations over which both ActivPal and Axivity monitors were recording data.

Supplementary Table 2: Summary table detailing the length of monitoring periods captured by both monitors simultaneously for T0, T1 and T2. The prosthesis used by each participant at each monitoring period is also noted. Note: Participants 4, 5, 6, 7 did not have access to a prosthesis at T0 so time shown is purely based of the Activpal data.

| **Participant** | **T0 Monitoring** | | | | **T1 Monitoring** | | | | **T2 Monitoring** | | | |
| --- | --- | --- | --- | --- | --- | --- | --- | --- | --- | --- | --- | --- |
|  | **Length of Recording (Activpal and Axivity)** | | | **Prosthesis** | **Length of Recording (Activpal and Axivity)** | | | **Prosthesis** | **Length of Recording (Activpal and Axivity)** | | | **Prosthesis** |
|  | **Days** | **Hours** | **Minutes** |  | **Days** | **Hours** | **Minutes** |  | **Days** | **Hours** | **Minutes** |  |
| P1 | 5 | 1 | 31 | Prior device | 7 | 23 | 2 | KOALAA | n/a | | | |
| P2 | 7 | 22 | 12 | Prior device | 7 | 1 | 44 | KOALAA | 7 | 0 | 0 | KOALAA |
| P3 | 7 | 4 | 43 | Prior device | 7 | 0 | 42 | KOALAA | 6 | 8 | 39 | Prior device |
| P4 | 6 | 16 | 34 | n/a | 6 | 8 | 4 | KOALAA | 3 | 10 | 0 | KOALAA |
| P5 | 7 | 11 | 43 | n/a | 7 | 1 | 1 | KOALAA | 0 | 8 | 47 | KOALAA |
| P6 | 9 | 11 | 59 | n/a | 8 | 19 | 55 | KOALAA | 4 | 0 | 1 | KOALAA |
| P7 | 6 | 6 | 16 | n/a | 6 | 1 | 35 | KOALAA | 7 | 0 | 0 | KOALAA |
| P8 | 7 | 1 | 54 | Prior device | 7 | 0 | 21 | KOALAA | 6 | 22 | 21 | Prior device |
